# Reprogramming of valine metabolism mediated by abnormally low ALDH6A1 expression promotes invasive metastasis of gastric cancer

**DOI:** 10.1126/sciadv.aeb2892

**Published:** 2026-07-08

**Authors:** Jipeng Wang, Lei Han, Gang Li, Hantao Guo, Wenyue Xu, Lianghui Xia, Menghan Nie, Qing Yang, Yinghao Luo, Yaxu Wang, Qiuming He, Yan-Xiao Ji, Bin Xiong, Shuyi Wang

**Affiliations:** ^1^ Department of Gastrointestinal Surgery, Zhongnan Hospital of Wuhan University, Wuhan 430071, China.; ^2^ Hubei Key Laboratory of Tumor Biological Behaviours, Wuhan 430071, China.; ^3^ Yunnan Cancer Hospital, The Third Affiliated Hospital of Kunming Medical University, Kunming, Yunnan, China.; ^4^ Department of Biological Repositories, Zhongnan Hospital of Wuhan University, Wuhan 430071, China.; ^5^ State Key Laboratory of Metabolism and Regulation in Complex Organisms, TaiKang Medical School (School of Basic Medical Sciences), Wuhan University, Wuhan 430071, China.; ^6^ Hubei Provincial Key Laboratory of Developmentally Originated Disease, Wuhan 430071, China.; ^7^ Department of Gastroenterology, The Central Hospital of Wuhan, Tongji Medical College, Huazhong University of Science and Technology, Wuhan, China.; ^8^ Department of Cardiology, Zhongnan Hospital of Wuhan University, Wuhan 430060, China.

## Abstract

Metastasis in gastric cancer requires metabolic reprogramming, but its key drivers remain unclear. Using a CRISPR-Cas9 metabolic knockout screen integrated with patient transcriptomes, we identified the mitochondrial enzyme ALDH6A1 as an anti-invasive factor. *ALDH6A1* down-regulation blocked the terminal step of valine catabolism and caused intracellular accumulation of methylmalonic acid (MMA). MMA competitively occupied the α-ketoglutarate (α-KG) cofactor pocket of the histone demethylase KDM5C, suppressing its activity and increasing H3K4 dimethylation (H3K4me2) at promoters of invasion-related genes, including *ANGPT2* (angiopoietin-2) and *MMP7* (matrix metalloproteinase 7). This epigenetic reprogramming promoted gastric cancer liver metastasis in mice. Pharmacologic ALDH6A1 activation with Alda-1 or systemic MMA clearance with l-carnitine lowered H3K4me2, dampened the invasive program, and reduced metastatic burden. These findings identify the ALDH6A1-MMA axis as a targetable metabolic-epigenetic pathway in gastric cancer metastasis.

## Teaser

Loss of ALDH6A1 drives MMA buildup that triggers epigenetic remodeling and promotes gastric cancer metastasis.

## INTRODUCTION

Tumor metastasis is a highly orchestrated, multistep cascade in which cancer cells breach the basement membrane and vasculature, survive as circulating tumor cells, seed distant niches as microscopic colonies, and ultimately expand into macroscopic lesions (*1*). Throughout this process, metabolic flexibility is indispensable: Dynamic intracellular rewiring and fluctuations in extracellular metabolites enable tumor cells to generate adenosine triphosphate (ATP) through aerobic glycolysis, sustain biosynthetic demands via hyperactive glutamine metabolism, and adapt to hostile conditions such as hypoxia, acidosis, and nutrient deprivation (*2*–*5*).

Gastric cancer ranks fifth worldwide for both incidence and cancer-related mortality (*6*), with metastatic dissemination representing the primary cause of death. Because local invasion is the initiating step of distant spread, targeting this process offers the most effective opportunity to suppress metastasis and improve patient outcomes. Multiomic analyses and large clinical cohorts have demonstrated that the extent of metabolic reprogramming is closely associated with tumor stage, recurrence risk, and prognosis in gastric cancer (*7*, *8*). Mechanistically, gastric cancer cells not only reroute nutrient uptake and catabolic pathways to meet the energetic demands of invasion (*9*) but also exploit metabolites as signaling molecules that directly activate pro-invasive pathways (*10*, *11*). However, a systematic delineation of the metabolic circuits driving invasion in gastric cancer remains lacking.

Here, we combined a genome-wide CRISPR-Cas9 metabolic knockout screen coupled with invasion-based selection in gastric cancer cells and integrated the resulting hits with patient transcriptomic data. This approach identified ALDH6A1 as a top anti-invasive effector. We demonstrate that silencing ALDH6A1 blocks the terminal step of valine degradation, resulting in intracellular accumulation of methylmalonic acid (MMA). MMA competitively binds α-ketoglutarate (α-KG) at the catalytic pocket of the histone demethylase (HDM) KDM5C, attenuating its enzymatic activity, increasing H3K4 dimethylation (H3K4me2) enrichment at the promoters of pro-invasive genes, and thus activating their transcription. Restoration of ALDH6A1 expression or scavenging of MMA, both in vitro and in mouse models, reduced promoter-associated H3K4me2 levels, suppressed the epithelial-mesenchymal transition (EMT), and markedly decreased liver colonization. Analyses of clinical samples revealed an inverse correlation between ALDH6A1 expression and serum MMA levels and linked low ALDH6A1 expression to poor prognosis.

Collectively, our findings delineate an ALDH6A1-MMA-KDM5C axis that links amino acid catabolism to chromatin regulation and metastatic fitness and suggest that metabolic activation of ALDH6A1 or depletion of MMA represents a tractable therapeutic strategy to prevent gastric cancer dissemination.

## RESULTS

### Identification of ALDH6A1 as a key regulatory target for gastric cancer invasion by CRISPR metabolic genomic library screening

**Fig. 1.**
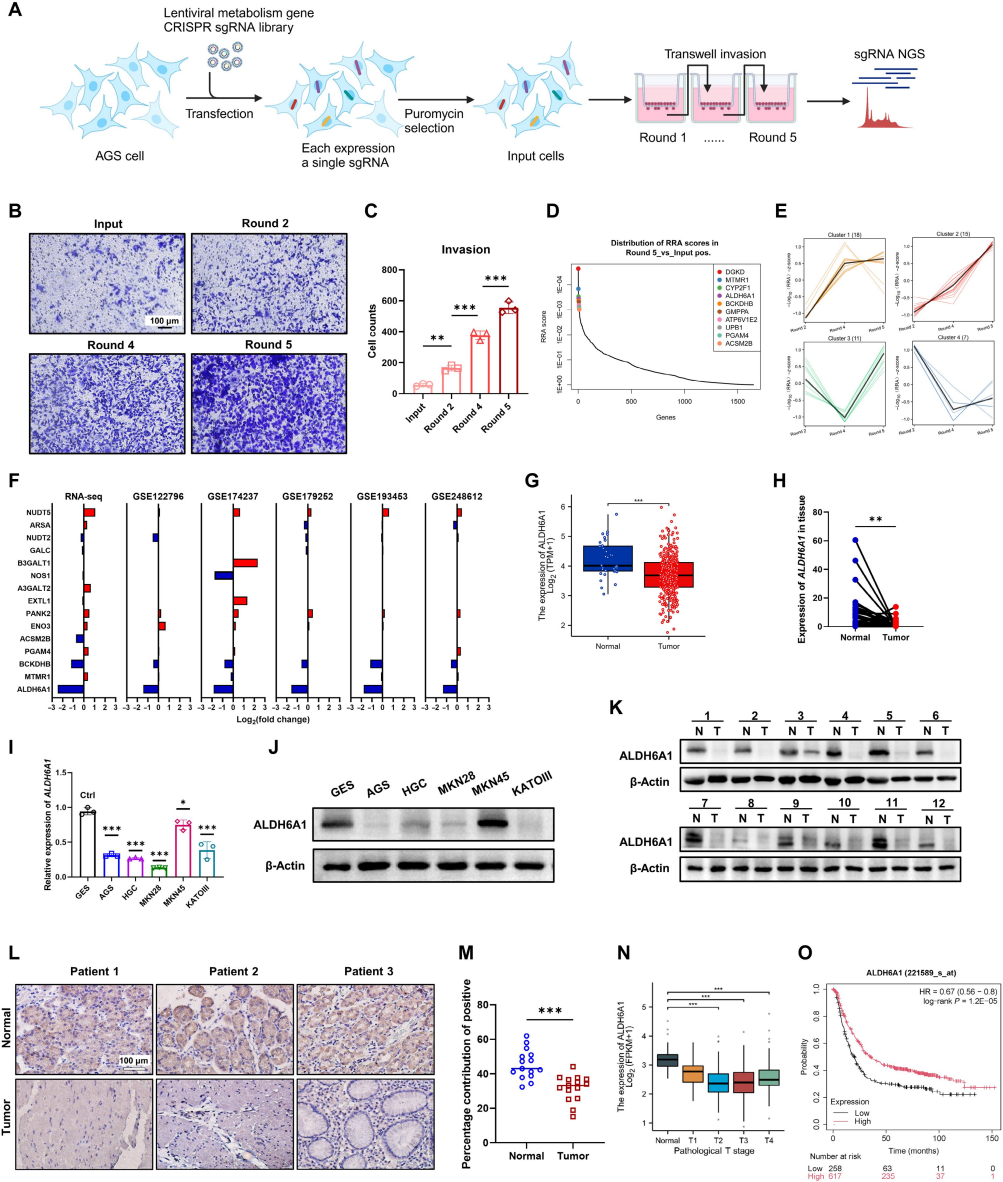
CRISPR metabolic screening identifies *ALDH6A1* as a suppressor of gastric cancer invasion. (**A**) Workflow of the in vitro CRISPR metabolic library screen under invasion selection pressure. NGS, next-generation sequencing. Created in BioRender. J. Wang (2026) https://BioRender.com/ce8r78e. (**B** and **C**) Representative Transwell invasion assays and quantification showing progressively increased AGS cell invasiveness after two, four, and five selection rounds (*n* = 3). Scale bar, 100 μm. (**D**) Metabolic genes ranked by MAGeCK RRA score, with the top 10 hits indicated. (**E**) *K*-means clustering showing the dynamic enrichment of invasion-associated genes across screening rounds. (**F**) Expression of progressively enriched Cluster 2 genes in paired gastric tumor and adjacent normal tissues from independent datasets. (**G**) ALDH6A1 mRNA abundance in TCGA-STAD tumors and normal tissues (tumor, *n* = 375; normal, *n* = 32). (**H**) RT-qPCR analysis of *ALDH6A1* in paired primary tumors and matched normal mucosa (*n* = 57 pairs). (**I** to **K**) ALDH6A1 expression in normal gastric epithelial and gastric cancer cell lines by RT-qPCR (I) and Western blotting (J) and in paired clinical samples by Western blotting (K; *n* = 12 pairs). (**L** and **M**) Representative ALDH6A1 IHC images and quantification in paired adjacent normal and tumor tissues (*n* = 15 pairs). Scale bar, 100 μm. (**N**) *ALDH6A1* expression [fragments per kilobase of transcript per million mapped reads (FPKM)] stratified by pathological T stage in TCGA-STAD. (**O**) Kaplan-Meier OS according to ALDH6A1 expression. HR, hazard ratio. Data are means ± SD. Statistical significance was determined by the two-tailed Student’s *t* test or one-way ANOVA with post hoc multiple comparisons. *P < 0.05, **P < 0.01, and ***P < 0.001.

To systematically investigate the metabolic reprogramming associated with gastric cancer invasion, we targeted 1724 metabolic genes in the AGS gastric cancer cell line using a metabolic gene knockout library containing 6896 sgRNAs at a multiplicity of infection (MOI) of <0.3. Through multiple rounds of in vitro Transwell invasion assays, we isolated subpopulations of cells with progressively increased invasiveness. These subpopulations were then subjected to high-throughput sequencing to identify key metabolic reprogramming events potentially driving gastric cancer invasion (Fig. 1A). In vitro results demonstrated that the invasiveness of AGS cell subpopulations increased gradually after two, four, and five rounds of screening (Fig. 1, B and C). Sequencing data were analyzed using the MAGeCK algorithm to calculate robust rank aggregation (RRA) scores for positively selected genes. Metabolic genes associated with gastric cancer invasion were identified using RRA < 0.05 and *P* < 0.05 as selection criteria. In the fifth round, 51 genes were significantly associated with gastric cancer invasion in the most invasive cell subpopulation (Fig. 1D). To identify genes progressively enriched over successive screening rounds, unsupervised *K*-means clustering was performed on these 51 differential genes. Among the four resulting clusters, 15 genes in Cluster 2 showed progressive enrichment with increasing screening rounds (Fig. 1E).

To account for potential human intervention in gene expression during the CRISPR library screening and to identify naturally occurring metabolic factors influencing invasion during gastric cancer progression, we integrated transcriptome data from patients with gastric cancer and Gene Expression Omnibus (GEO) datasets (GSE122796, GSE174237, GSE179252, GSE193453, and GSE248612), comparing cancerous and adjacent noncancerous tissues to validate the 15 previously identified genes. Analysis revealed that the aldehyde dehydrogenase family member *ALDH6A1* was consistently underexpressed in gastric cancer tissues across all datasets and exhibited the lowest expression levels (Fig. 1F). Consequently, subsequent studies would focus on ALDH6A1. To investigate whether ALDH6A1 contributes to the invasive progression of gastric cancer, we first examined its expression in gastric cancer versus normal tissues using The Cancer Genome Atlas (TCGA) database. *ALDH6A1* was significantly underexpressed in gastric cancer tissues (Fig. 1G), a finding further validated in paired gastric cancer and adjacent noncancerous tissues, as well as in cell lines (Fig. 1, H to M). Analysis of the relationship between *ALDH6A1* expression and clinicopathological stage in the TCGA STAD database revealed that lower expression was associated with higher T stage in patients with gastric cancer (Fig. 1N). Survival curves plotted using the Kaplan-Meier Plotter (www.kmplot.com) also indicated that low *ALDH6A1* expression correlated with poorer overall survival (OS) in patients with gastric cancer (Fig. 1O).

Collectively, the CRISPR screen, multicohort validation, and clinical associations converge on ALDH6A1 as a central metabolic suppressor of gastric cancer invasion.

### ALDH6A1 enzymatic activity constrains gastric cancer invasion and migration

**Fig. 2.**
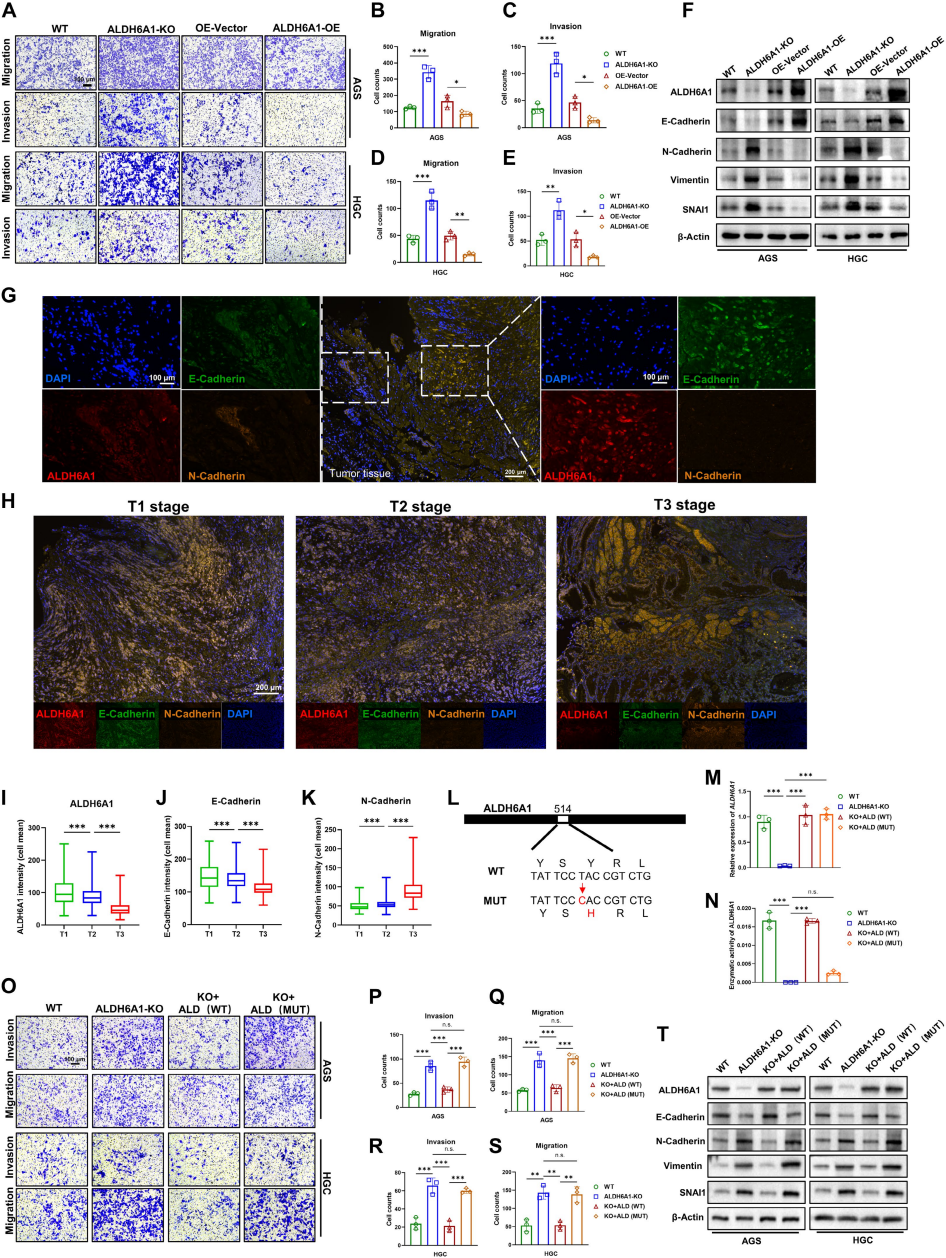
Catalytic activity of ALDH6A1 constrains invasion in gastric cancer. (**A**) Representative images of Transwell migration and Matrigel invasion assays in AGS and HGC-27 cells (WT, ALDH6A1-KO, OE-vector, and ALDH6A1-OE). Scale bar, 100 μm. (**B** and **C**) Quantification of migrated (B) and invaded (C) AGS cells from (A). (**D** and **E**) Quantification of migrated (D) and invaded (E) HGC-27 cells from (A). (**F**) Western blot analysis was used to assess canonical EMT markers in the same cells. (**G**) mIHC of matched tumor cores and invasive fronts revealed reciprocal distribution of ALDH6A1 and EMT markers. Scale bars, 200 μm for the overview image and 100 μm for magnified images. (**H**) Representative multiplex immunofluorescence images showing ALDH6A1 (red), E-cadherin (green), N-cadherin (orange), and DAPI (blue) in gastric cancer tissues across T1, T2, and T3 stages. Scale bar, 200 μm. (**I** to **K**) Quantification of mean fluorescence intensity (cell mean) for ALDH6A1 (I), E-cadherin (J), and N-cadherin (K) from images in (H). (**L**) Schematic of the catalytically inactive ALDH6A1-MUT. (**M** and **N**) mRNA levels (M) and enzymatic activity (N) of ALDH6A1-WT or ALDH6A1-MUT in AGS (ALDH6A1-KO) cells (*n* = 3). (**O**) Representative Transwell migration and Matrigel invasion images of AGS and HGC-27 cells, including WT, ALDH6A1-KO, and KO cells rescued with WT ALDH6A1 [KO+ALD (WT)] or a catalytically inactive mutant [KO+ALD (MUT)]. (**P** and **Q**) Quantification of invaded (P) and migrated (Q) AGS cells from (O). (**R** and **S**) Quantification of invaded (R) and migrated (S) HGC-27 cells from (O). (**T**) Western blot analysis showing that rescue with WT ALDH6A1, but not catalytically inactive ALDH6A1-MUT, restored E-cadherin expression and suppressed N-cadherin and vimentin. Data are means ± SD. Statistical significance was assessed by the one-way ANOVA with post hoc multiple comparisons. *P < 0.05, **P < 0.01, and ***P < 0.001; n.s., not significant.

To further validate the effects of ALDH6A1 expression on the invasion and migration of gastric cancer cells, we generated *ALDH6A1* overexpression (ALDH6A1-OE) and *ALDH6A1* knockout (ALDH6A1-KO) cell lines and assessed their invasive and migratory abilities using Transwell assays. The results demonstrated that deletion of ALDH6A1 significantly enhanced the invasive and migratory abilities of gastric cancer cells, whereas overexpression of ALDH6A1 inhibited these abilities (Fig. 2, A to E). The EMT plays a key role in tumor invasion, during which tumor cells lose epithelial characteristics and acquire mesenchymal properties, thus gaining an invasive phenotype (*12*). To investigate whether ALDH6A1 regulates gastric cancer cell invasion through the EMT, we examined the expression of EMT-related markers in ALDH6A1-OE and ALDH6A1-KO cell lines using Western blotting. ALDH6A1-KO significantly decreased the epithelial marker E-cadherin, increased the expression levels of the mesenchymal markers N-cadherin and vimentin, and promoted the EMT in gastric cancer cells, whereas ALDH6A1-OE inhibited the EMT (Fig. 2F). Because the tumor border zone is typically the most active area for invasion and metastasis (*13*), we assessed the expression levels of ALDH6A1 and EMT markers in the border and core zones of gastric cancer tissues via multiplex immunohistochemistry (mIHC). The results showed that, compared with the tumor core, cells in the marginal zone exhibited a more pronounced EMT phenotype accompanied by lower ALDH6A1 expression (Fig. 2G and fig. S3). Further analysis of ALDH6A1 expression and EMT markers in tumor tissues from patients with gastric cancer across different T stages revealed that ALDH6A1 levels progressively decreased with increasing T stage, whereas the EMT phenotype was significantly enhanced (Fig. 2, H to K). These findings suggest that reduced ALDH6A1 expression may promote gastric cancer invasion by facilitating the EMT in tumor cells.

As a key rate-limiting enzyme in valine metabolism, ALDH6A1 can regulate metabolism through its classical enzyme function (*14*) as well as affect downstream molecules through nonclassical protein interactions (*15*). To determine whether ALDH6A1-mediated regulation of the EMT and invasive metastasis in gastric cancer depends on its enzymatic activity, we constructed the plasmid mutant ALDH6A1 (ALDH6A1-MUT) (c.514T>C, p.Tyr172His [Y172H]) (Fig. 2L), which harbors a mutation that abolishes the ALDH6A1 enzymatic activity, and performed rescue experiments in ALDH6A1-KO cells. The results showed that the mutation at the c.514T>C substitution did not affect ALDH6A1 expression levels (Fig. 2M) but significantly reduced its enzymatic activity (Fig. 2N). The invasive and migratory abilities of gastric cancer cells were further assessed using the Transwell assay. The results showed that reexpression of wild-type ALDH6A1 (ALDH6A1-WT) was able to significantly attenuate the enhanced invasiveness of ALDH6A1-KO cells, consistent with previous findings. In contrast, reexpression of ALDH6A1-MUT failed to reduce the heightened invasiveness induced by ALDH6A1-KO (Fig. 2, O to S). Examination of EMT phenotypes revealed that only ALDH6A1-WT reexpression markedly suppressed the EMT in gastric cancer cells, whereas the mutant had no significant effect (Fig. 2T). These results indicate that the increased invasiveness observed after ALDH6A1-KO is primarily due to loss of its enzymatic activity rather than its nonenzymatic functions.

### Deficiency of ALDH6A1 in gastric cancer mediates the accumulation of MMA, a by-product of valine metabolism

**Fig. 3.**
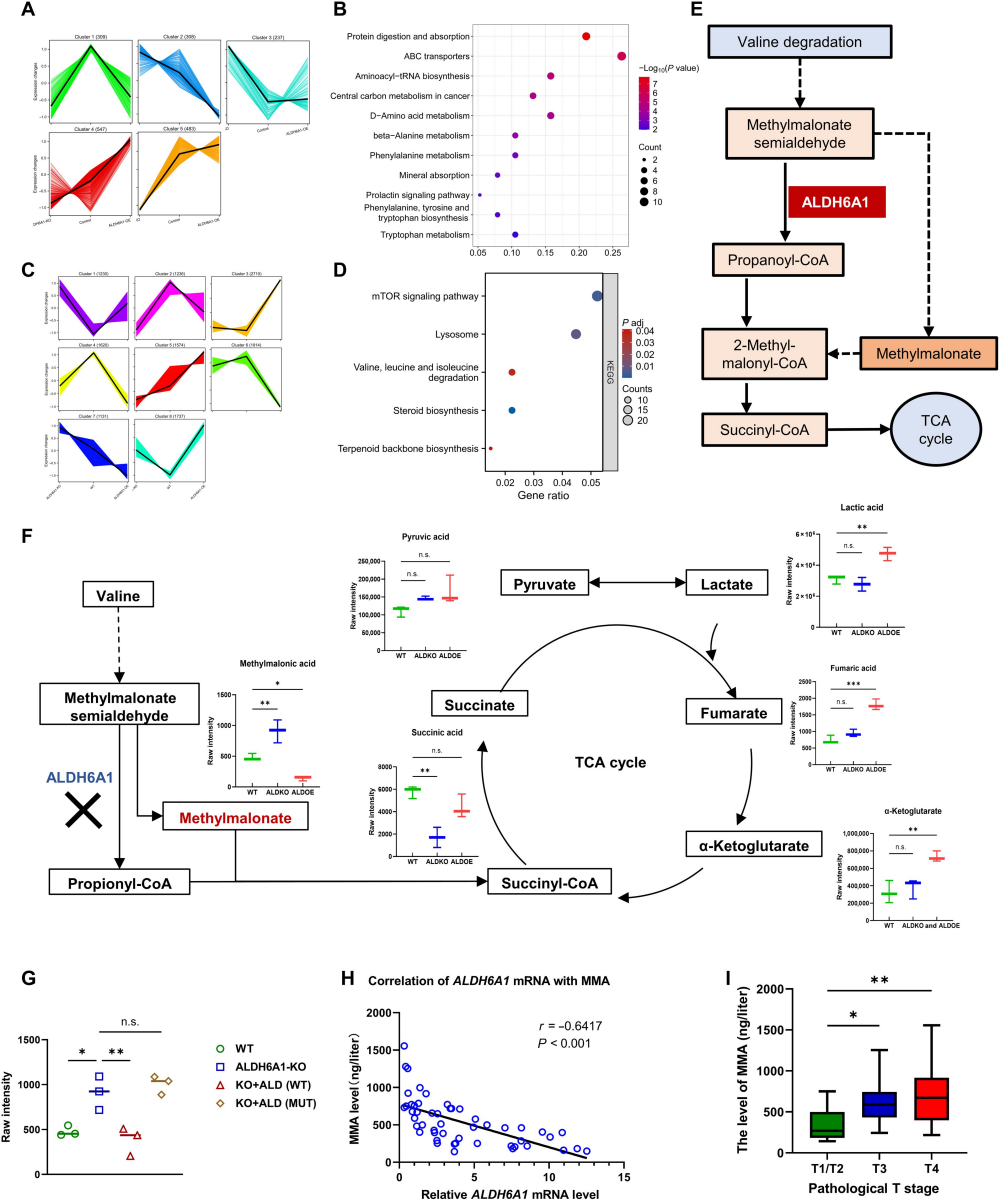
Loss of ALDH6A1 drives MMA accumulation by rewiring valine catabolism. (**A**) Unbiased *K*-means clustering of untargeted metabolomics profiles from AGS cells with ALDH6A1-KO (KO), WT control (WT), or ALDH6A1-OE (OE) (*n* = 3 per group). (**B**) KEGG enrichment analysis of metabolite Clusters 2 and 4, which varied with ALDH6A1 expression; the top 10 enriched pathways are shown. (**C**) *K*-means clustering of RNA-seq data from the same cells. (**D**) KEGG enrichment analysis of gene Clusters 5 and 7, which varied with ALDH6A1 expression. (**E**) Schematic of the valine degradation pathway highlighting the ALDH6A1-catalyzed step. (**F**) Schematic of the valine catabolism linked to the TCA cycle, annotated with fold changes of the indicated organic acids. Metabolite abundances were measured by targeted LC-MS/MS organic acid quantification (*n* = 3 per group). (**G**) LC-MS/MS quantification of intracellular MMA after rescue with ALDH6A1-WT or catalytic-dead ALDH6A1-MUT in ALDH6A1-KO cells (*n* = 3). (**H**) Inverse correlation between *ALDH6A1* mRNA abundance in tumors and serum MMA levels in patients with gastric cancer (*n* = 50). (**I**) Serum MMA levels stratified by pathological T stage (T1/T2, *n* = 18; T3, *n* = 17; T4, *n* = 15). Data are means ± SD. Statistical significance was assessed by the two-tailed Student’s *t* test or one-way ANOVA with post hoc multiple comparisons, and correlations were analyzed by simple linear regression. *P < 0.05, **P < 0.01, and ***P < 0.001; n.s., not significant.

ALDH6A1, as a metabolic enzyme, primarily catalyzes the dehydrogenation of aldehyde metabolites, particularly in β-alanine and valine metabolism. Given that its regulatory role in gastric cancer invasion is largely dependent on its enzymatic activity, we focused on the metabolic alterations induced by changes in ALDH6A1 expression. Untargeted metabolomic profiling of ALDH6A1-OE and ALDH6A1-KO cell lines was performed to explore the metabolic pathways affected by ALDH6A1 in gastric cancer. *K*-means unsupervised clustering of the altered metabolites identified five clusters, among which 308 metabolites in Cluster 2 decreased as ALDH6A1 expression increased, whereas 547 metabolites in Cluster 4 increased with an increase in ALDH6A1 expression (Fig. 3A). Thus, we hypothesized that changes in metabolites in Clusters 2 and 4 were directly linked to ALDH6A1 expression. Kyoto Encyclopedia of Genes and Genomes (KEGG) pathway enrichment analysis of metabolites in Clusters 2 and 4 revealed that the “central carbon metabolism” and “amino acid metabolism” were significantly enriched (Fig. 3B). Integrating these findings with transcriptomic data, all altered genes were classified into eight clusters by *K*-means unsupervised clustering. Among these, 1574 genes in Cluster 5 were positively correlated with ALDH6A1 expression, whereas 1131 genes in Cluster 7 were negatively correlated (Fig. 3C). KEGG pathway enrichment analysis of genes in Clusters 5 and 7 identified multiple metabolism-associated pathways [e.g., lysosome, mechanistic target of rapamycin (mTOR) signaling, and steroid metabolism; Fig. 3D]. Notably, “valine, leucine and isoleucine degradation” was highlighted because ALDH6A1 is a direct enzymatic component of this pathway.

Valine, a branched-chain amino acid, is metabolized to MMA semialdehyde upon entering the cell, which is subsequently converted to propionyl–coenzyme A (CoA) by ALDH6A1 and ultimately enters the tricarboxylic acid (TCA) cycle as succinyl CoA. Concurrently, MMA semialdehyde can be shunted through a bypass pathway to generate MMA (Fig. 3E). MMA has been reported to promote tumor invasion and metastasis (*16*–*18*). To determine whether ALDH6A1 deletion leads to MMA accumulation in gastric cancer cells, we performed targeted organic acid metabolomics analysis on ALDH6A1-KO cell lines. The results showed that, among the detectable organic acid metabolites, only MMA levels were increased (Fig. 3F). Further analysis using liquid chromatography–tandem mass spectrometry (LC-MS/MS) in gastric cancer cells reexpressing ALDH6A1-WT and ALDH6A1-MUT demonstrated that restoration of WT ALDH6A1 significantly reduced MMA levels, whereas expression of the mutant form failed to alleviate MMA accumulation (Fig. 3G). Under [U-^13^C_5_]valine tracing conditions, AGS ALDH6A1-KO cells exhibited a markedly increased M+3 fraction and a decreased M+0 fraction of MMA compared with WT cells (fig. S11B), indicating that more valine-derived labeled carbon was aberrantly redirected toward MMA accumulation. These findings were validated in clinical gastric cancer samples, revealing a negative correlation between *ALDH6A1* expression in gastric cancer tissues and serum MMA levels (Fig. 3H) and showing that serum MMA levels progressively increased with advancing T stage (Fig. 3I).

These results suggest that deletion of ALDH6A1 leads to abnormal valine metabolism in gastric cancer cells, which, in turn, triggers the accumulation of the metabolic by-product MMA, a process that may be a key mechanism by which ALDH6A1 regulates gastric cancer cell invasion.

### ALDH6A1-mediated MMA accumulation promotes histone H3K4me2 modification in gastric cancer cells

**Fig. 4.**
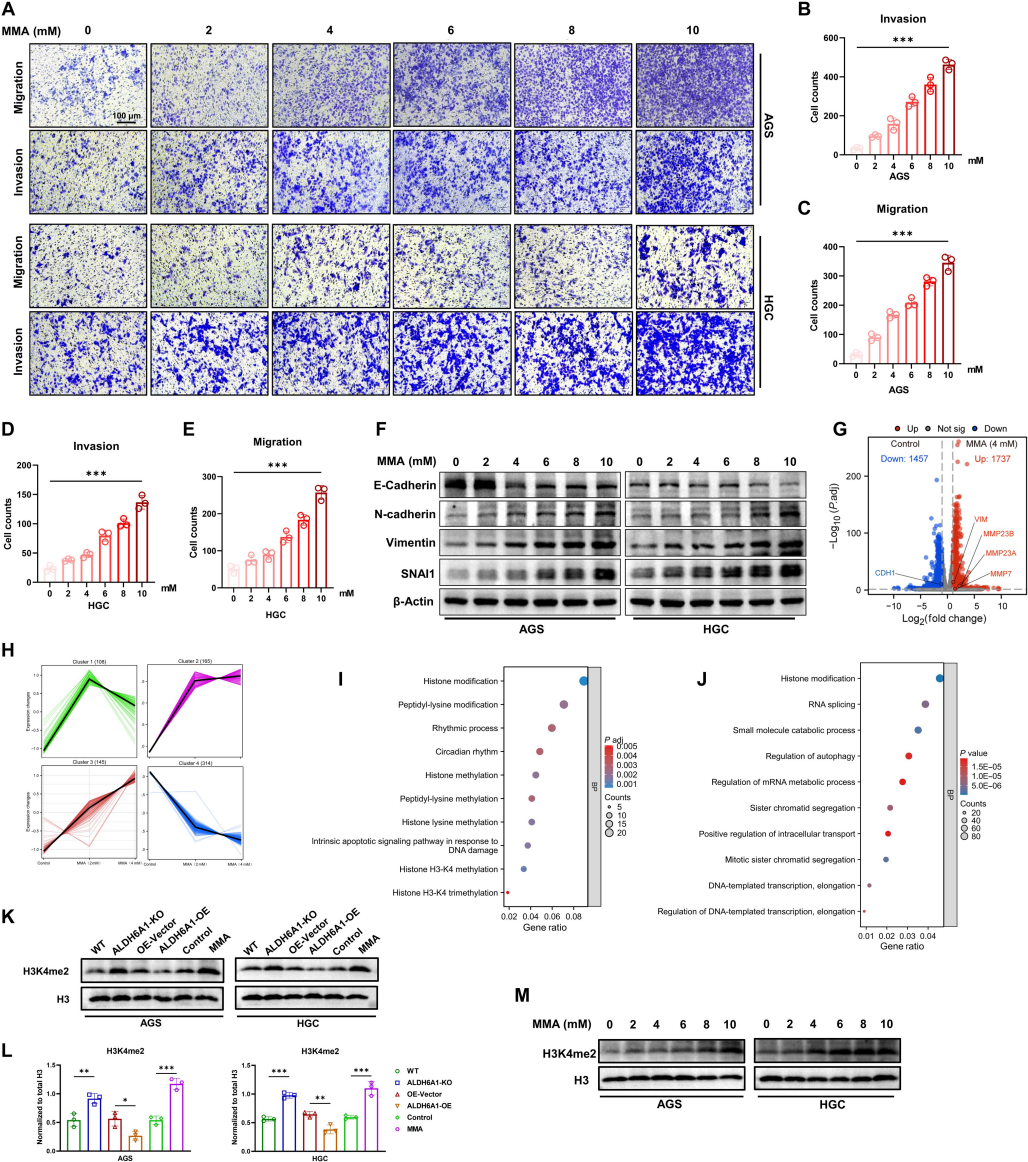
MMA accumulation downstream of ALDH6A1 deficiency enhances gastric cancer invasion by increasing H3K4me2. (**A** to **E**) Representative Transwell migration and Matrigel invasion images (A) and quantification of invaded and migrated AGS (B and C) and HGC-27 cells (D and E) after treatment with increasing MMA concentrations (0, 2, 4, 6, 8, and 10 mM) for 10 days. Scale bar, 100 μm. (**F**) Western blot analysis of EMT markers in the same cells. (**G**) Volcano plots of RNA-seq changes after 4 mM MMA treatment for 10 days (*n* = 3); differentially expressed genes were defined by *P* < 0.05 and |log_2_FC| > 1. (**H** and **I**) *K*-means clustering of transcriptomes across the MMA gradient (0, 2, and 4 mM; *n* = 3 per group) and GO enrichment of MMA-responsive Clusters 3 and 4. (**J**) GO enrichment of ALDH6A1-responsive Clusters 5 and 7 from Fig. 3C. (**K** and **L**) Western blot and densitometric quantification of H3K4me2, normalized to total H3, in AGS and HGC-27 cells under the indicated ALDH6A1-KO, ALDH6A1-OE, vehicle control, and 4 mM MMA conditions. (**M**) Dose-dependent induction of H3K4me2 in AGS cells treated with 0 to 10 mM MMA. Data are means ± SD. Statistical significance was assessed by the one-way ANOVA with post hoc multiple comparisons. *P < 0.05, **P < 0.01, and ***P < 0.001.

To determine whether low ALDH6A1 expression promotes gastric cancer cell invasion via the valine metabolism by-product MMA, we cultured gastric cancer AGS and HGC-27 cells for 10 days with increasing concentrations of MMA (0 to 10 mM), and cellular phenotypes were monitored. The Transwell assay showed that the invasion and migration ability of AGS and HGC-27 cells were dose-dependently increased with increasing MMA concentration (Fig. 4, A to E). Moreover, the cells gradually changed from the epithelial cell phenotype to the mesenchymal cell phenotype (Fig. 4F). To further investigate the molecular mechanisms by which MMA promotes gastric cancer cell invasion, we performed transcriptome sequencing of AGS cells treated with low-dose (2 mM) and high-dose (4 mM) MMA. High-dose MMA treatment significantly up-regulated multiple invasion-associated genes, including members of the *VIM* and *MMP* families, and down-regulated the epithelial marker *CDH1* (Fig. 4G). Genes exhibiting expression changes in response to increasing MMA concentrations were further analyzed by *K*-means unsupervised clustering. Genes in Cluster 3 exhibited increased expression with increasing MMA concentration, whereas genes in Cluster 4 showed decreased expression as the MMA concentration increased (Fig. 4H). GO enrichment analysis of Clusters 3 and 4 revealed that the changed genes were primarily involved in histone modifications, particularly histone H3K4 methylation (Fig. 4I). Because MMA accumulation in gastric cancer cells is mediated by low ALDH6A1 expression, we further analyzed transcriptome sequencing data from ALDH6A1-KO and ALDH6A1-OE cells by performing KEGG enrichment on Clusters 5 and 7, which contain genes whose expression changes with elevated ALDH6A1 levels. The results also indicated that changes in ALDH6A1 expression could influence histone modifications (Fig. 4J). To further investigate MMA-mediated histone methylation changes, we performed Western blotting to examine six types of histone methylation in gastric cancer cells with altered ALDH6A1 expression or MMA treatment (fig. S8). The results showed that H3K4me2 was affected: H3K4me2 increased with ALDH6A1-KO, decreased with ALDH6A1-OE, and increased upon MMA treatment (Fig. 4, K and L), suggesting that MMA specifically triggers H3K4me2 modifications. Dose-gradient experiments with MMA further confirmed that H3K4me2 levels gradually increased in a concentration-dependent manner (Fig. 4M).

Together, we can conclude that MMA accumulation mediated by low ALDH6A1 expression in gastric cancer cells regulates gastric cancer cell invasion by promoting histone H3K4me2 modification.

### MMA regulates histone H3K4me2 modification of the gastric cancer invasion-related gene promoter to promote its expression

**Fig. 5.**
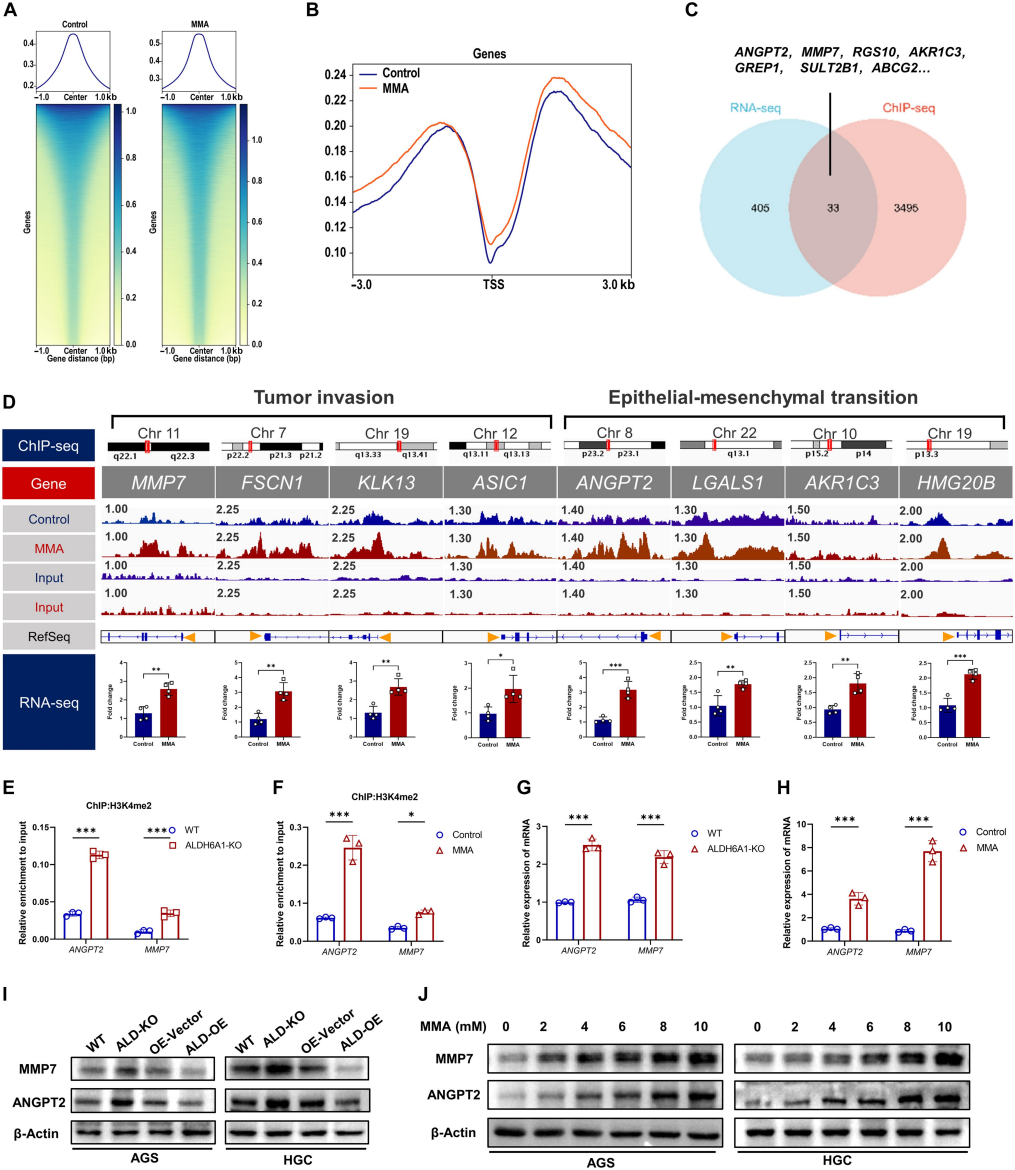
MMA enhances H3K4me2 at the *MMP7* and *ANGPT2* promoters to up-regulate their expression. (**A** and **B**) Heatmaps (A) and average profiles (B) of H3K4me2 ChIP-seq signals within ±1 kb (A) or ± 3 kb (B) of TSSs are shown for control and MMA-treated AGS cells (4 mM, 10 days). (**C**) Venn diagram intersecting genes significantly up-regulated by RNA-seq (*P* < 0.05, log_2_FC ≥ 1) with genes exhibiting increased H3K4me2 after MMA treatment. (**D**) Integrative Genomics Viewer tracks illustrating H3K4me2 enrichment (red, MMA; blue, control) around the *MMP7* and *ANGPT2* promoters; bar charts on the right show the corresponding mRNA fold changes. (**E** and **F**) ChIP-qPCR confirms elevated H3K4me2 at the *ANGPT2* and *MMP7* promoters after ALDH6A1-KO (E) or MMA exposure (F) (*n* = 3). (**G** and **H**) RT-qPCR showing that ALDH6A1 loss (G) or MMA treatment (H) up-regulates *ANGPT2* and *MMP7* transcripts (*n* = 3). (**I**) Western blot analysis demonstrating changes in ANGPT2 and MMP7 proteins after ALDH6A1-KO (KO) or ALDH6A1-OE (OE). (**J**) Dose-dependent induction of ANGPT2 and MMP7 proteins by increasing MMA concentrations. Data are means ± SD. Statistical significance was assessed by the two-tailed Student’s *t* test. *P < 0.05, **P < 0.01, and ***P < 0.001.

Methylation of the H3K4 locus is one of the most widely studied histone modifications, and elevated dimethylation or trimethylation at gene promoters is generally associated with transcriptional activation of oncogenes (*19*). To determine whether MMA-induced increases in H3K4me2 drive the expression of gastric cancer invasion-related genes, we performed chromatin immunoprecipitation sequencing (ChIP-seq) for H3K4me2 in AGS cells treated with 4 mM MMA. The results demonstrated substantial enrichment of H3K4me2 near transcription start sites (TSSs) following MMA treatment (Fig. 5, A and B), indicating that MMA enhances promoter H3K4me2 levels in gastric cancer cells, thus promoting the transcription of target genes. To identify potential downstream effector genes, we jointly analyzed the RNA sequencing (RNA-seq) and ChIP-seq data from MMA-treated AGS cells. Differentially up-regulated genes from RNA-seq [*P* < 0.05, log_2_(fold change) (log_2_FC) ≥ 1] were intersected with genes exhibiting increased H3K4me2 modification in ChIP-seq to identify targets whose transcription was likely activated by MMA-mediated H3K4me2 modification. This analysis yielded 33 genes (Fig. 5C), including both direct promoters of tumor invasion (*MMP7*, *FSCN1*, *KLK13*, and *ASIC1*) and known EMT regulators (*ANGPT2*, *LGALS1*, *AKR1C3*, and *HMG20B*), suggesting that elevated H3K4me2 levels at their promoters drive their increased expression (Fig. 5D).

Matrix metalloproteinase 7 (MMP7) is an important member of the matrix MMP family, which can promote tumor invasion by degrading the extracellular matrix (*20*) and mediate the EMT (*21*). Angiopoietin-2 (ANGPT2), a member of the angiopoietin family, regulates angiogenesis, vascular remodeling, and inflammatory responses through interaction with the Tie2 receptor (*22*), which is also capable of regulating EMT processes in tumors (*23*). Given the critical roles of both genes in tumor invasion and the EMT, they were selected for subsequent functional validation. ChIP–quantitative polymerase chain reaction (qPCR) confirmed that H3K4me2 levels in the promoter regions of *MMP7* and *ANGPT2* were elevated in ALDH6A1-KO gastric cancer cells (Fig. 5E), consistent with changes observed in MMA-treated gastric cancer cells (Fig. 5F). Reverse transcription qPCR (RT-qPCR) and Western blot analyses further demonstrated that ALDH6A1 deletion or MMA treatment dose-dependently increased both mRNA and protein levels of these genes (Fig. 5, G to J).

These findings suggest that low *ALDH6A1* expression leads to MMA accumulation, which promotes gastric cancer invasion by enhancing the H3K4me2 modification of the promoters of key genes for an invasive EMT, such as *MMP7* and *ANGPT2*, and driving their expression.

### MMA regulates gastric cancer histone H3K4me2 modification by inhibiting demethylase KDM5C activity

**Fig. 6.**
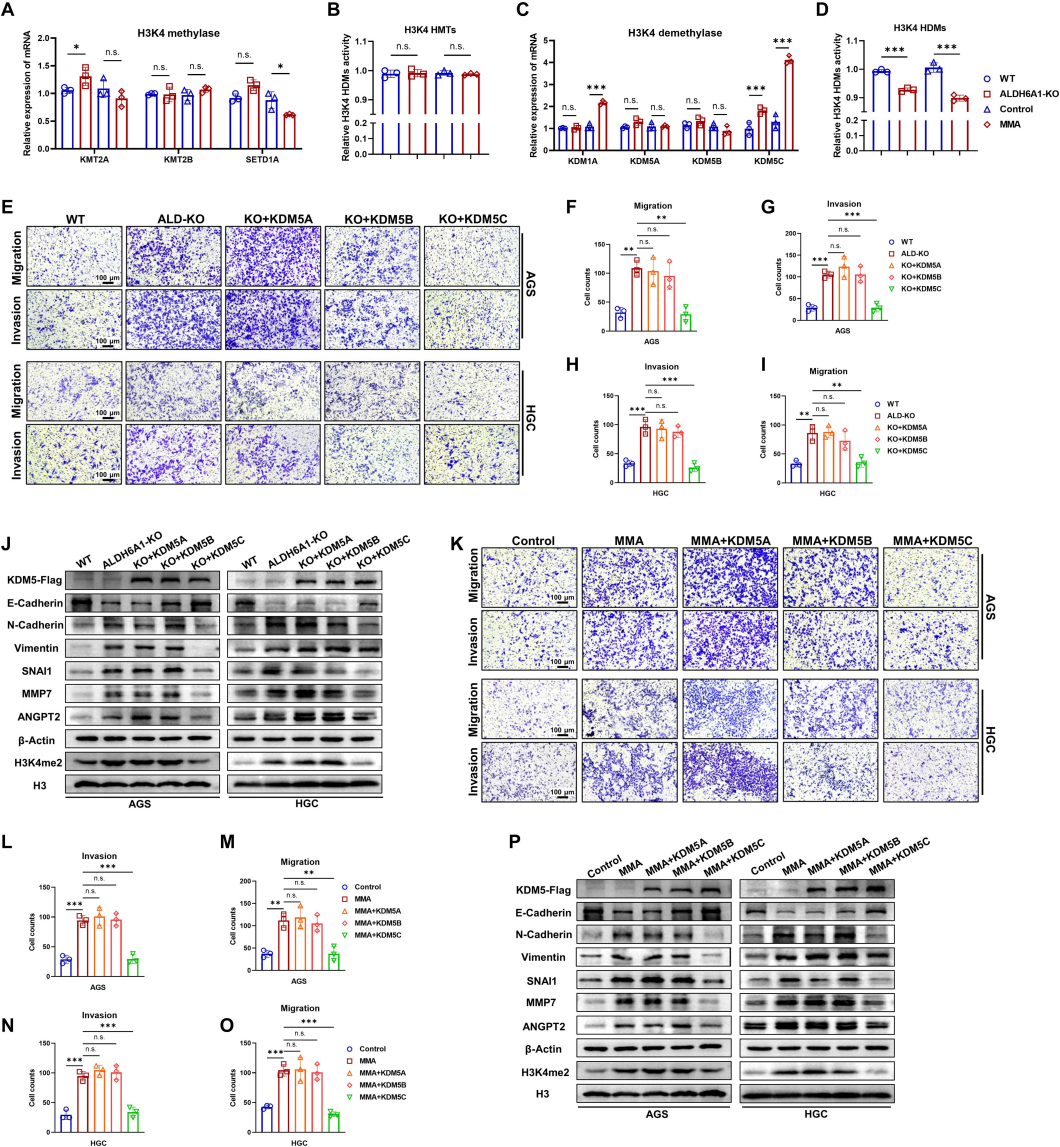
MMA suppresses KDM5C activity, elevates H3K4me2, and drives gastric cancer invasion. (**A**) RT-qPCR analysis of representative H3K4 histone methyltransferases (H3K4 HMTs; *KMT2A*, *KMT2B*, and *SETD1A*) in AGS cells with ALDH6A1-KO or MMA treatment (10 mM, 10 days) (*n* = 3). (**B**) Global H3K4 HMT activity under the same conditions (*n* = 3). (**C**) RT-qPCR analysis of H3K4 HDMs (*KDM1A*, *KDM5A*, *KDM5B*, and *KDM5C*). (**D**) Global H3K4 HDM activity under the same conditions (*n* = 3). (**E** to **I**) Representative Transwell migration and Matrigel invasion images (E) and quantification of invaded and migrated AGS (F and G) and HGC-27 cells (H and I) in WT, ALDH6A1-KO, and ALDH6A1-KO cells reexpressing KDM5A, KDM5B, or KDM5C. Scale bars, 100 μm. (**J**) Western blot analysis of EMT markers and H3K4me2 in the same cells. (**K** to **O**) Representative Transwell migration and Matrigel invasion images (K) and quantification of invaded and migrated AGS (L and M) and HGC-27 cells (N and O) after MMA treatment with reexpression of KDM5A, KDM5B, or KDM5C. Scale bars, 100 μm. (**P**) Western blot analysis showing that KDM5C, but not KDM5A or KDM5B, reduced EMT markers and H3K4me2 in MMA-treated cells. Data are means ± SD. Statistical significance was determined by the one-way ANOVA with post hoc multiple comparisons. *P < 0.05, **P < 0.01, and ***P < 0.001.

Homeostasis of histone H3K4 methylation is maintained by the dynamic balance between histone methyltransferase (HMT) and HDM (*24*). To elucidate the mechanisms underlying MMA-induced H3K4me2 elevation, we examined the transcriptional and enzymatic status of key H3K4 HMTs (*KMT2A*, *KMT2B*, and *SETD1A*) and HDMs (*KDM1A*, *KDM5A*, *KDM5B*, and *KDM5C*). Neither ALDH6A1 deletion nor MMA treatment altered the mRNA expression or catalytic activity of the H3K4 HMTs in AGS cells (Fig. 6, A and B). Among the HDMs, only *KDM5C* transcription was up-regulated, accompanied by a significant decrease in total H3K4-HDM activity (Fig. 6, C and D), suggesting that MMA may enrich H3K4me2 levels by inhibiting KDM5C activity.

Subsequently, KDM5A, KDM5B, and KDM5C were individually overexpressed in ALDH6A1-KO cells. Only KDM5C overexpression significantly attenuated the enhanced invasion and migration induced by ALDH6A1 deletion (Fig. 6, E to I), suppressed the EMT, and reduced H3K4me2 levels (Fig. 6J). Similarly, in MMA-treated cells, restoration of KDM5C alone reversed MMA-induced invasion and H3K4me2 accumulation (Fig. 6, K to P).

Collectively, these results indicate that low *ALDH6A1* expression leads to MMA accumulation, which promotes gastric cancer cell invasion by inhibiting the demethylase activity of KDM5C and enriching H3K4me2 at promoter regions, thus activating invasion-related transcriptional programs.

### MMA promotes gastric cancer invasive migration by inhibiting the demethylation activity of KDM5C through competitive binding with α-KG

KDM5 proteins are Jumonji C (JmjC) domain-containing members of the α-KG–dependent dioxygenase superfamily, and their catalytic activity requires the coordinated participation of oxygen, Fe^2+^, and α-KG (*25*). Previous studies have shown that organic acids such as succinate, fumarate, and 2-hydroxyglutarate can inhibit these dioxygenases by competing with α-KG for its binding site (*26*–*28*). Given that MMA is structurally and physicochemically similar to succinate (*29*), we hypothesize that MMA can also competitively occupy the α-KG binding site and thus inhibit KDM5C demethylation.

**Fig. 7.**
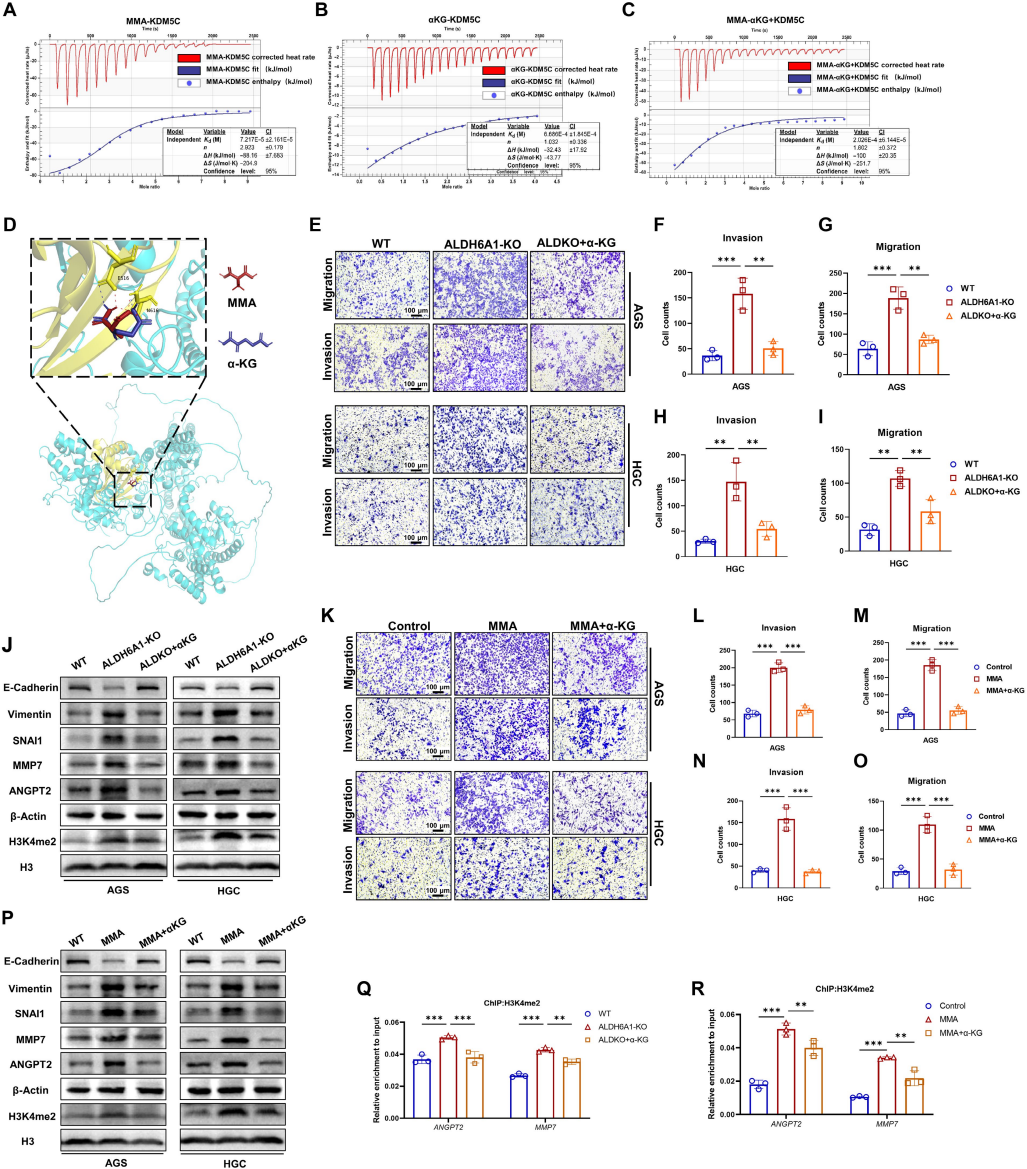
MMA competes with α-KG for KDM5C, inhibits demethylase activity, and enriches H3K4me2 to potentiate gastric cancer invasion. (**A** to **C**) ITC analysis of KDM5C binding to MMA (A), α-KG (B), and a premixed MMA + α-KG solution (C). MMA bound KDM5C with a higher affinity (*K*_d_ = 7.22 × 10^−5^ M) than α-KG (*K*_d_ = 6.69 × 10^−4^ M), and the presence of MMA altered the apparent α-KG–binding profile (*K*_d_ = 2.03 × 10^−4^ M). CI, confidence interval. (**D**) Docking model based on the AlphaFold 3 showing the overlap of MMA and α-KG within the KDM5C catalytic pocket (E516 and N616). (**E** to **I**) Representative Transwell migration and Matrigel invasion images (E) and quantification of invaded and migrated AGS (F and G) and HGC-27 cells (H and I) in WT, ALDH6A1-KO, and ALDH6A1-KO cells supplemented with α-KG (5 mM, 10 days). Scale bars, 100 μm. (**J**) Western blot analysis showing that α-KG supplementation reversed the enhanced EMT and H3K4me2 signature induced by ALDH6A1-KO. (**K** to **O**) Representative Transwell migration and Matrigel invasion images (K) and quantification of invaded and migrated AGS (L and M) and HGC-27 cells (N and O) treated with MMA (10 mM) in the absence or presence of α-KG supplementation (5 mM) for 10 days. Scale bars, 100 μm. (**P**) Western blot analysis showing that α-KG abolished the pro-invasive phenotype and H3K4me2 accumulation induced by MMA. (**Q** and **R**) ChIP-qPCR analysis showing that α-KG reduced H3K4me2 enrichment at the *ANGPT2* and *MMP7* promoters in ALDH6A1-KO and MMA-treated cells (*n* = 3). Data are means ± SD. Statistical significance was determined by one-way ANOVA with post hoc multiple comparisons for (F) to (I) and (L) to (O) and by two-way ANOVA for (Q) and (R). **P < 0.01 and ***P < 0.001.

To test this hypothesis, we first used isothermal titration calorimetry (ITC) to measure the binding affinity of MMA to KDM5C. The equilibrium dissociation constant for MMA (*K*_d_ = 7.2 × 10^−5^ M; Fig. 7A) was substantially lower than that of α-KG binding to KDM5C (*K*_d_ = 6.7 × 10^−4^ M; Fig. 7B). Furthermore, in the presence of MMA, α-KG showed an altered apparent binding profile with faster saturation (*K*_d_ = 2.0 × 10^−4^ M; Fig. 7C), indicating direct competition between the two ligands. Molecular docking based on the AlphaFold 3–predicted KDM5C structure further revealed that MMA and α-KG occupy overlapping binding sites within the catalytic region of KDM5C (E516 and N616) (Fig. 7D).

At the functional level, supplementation with 5 mM α-KG for 10 days in ALDH6A1-deficient gastric cancer cells significantly reversed the enhanced invasion and migration (Fig. 7, E to I) and reduced EMT markers and H3K4me2 levels (Fig. 7J). Similarly, exogenous α-KG counteracted the invasion-promoting effects and H3K4me2 accumulation induced by MMA treatment (Fig. 7, K to P). ChIP-qPCR further confirmed that α-KG supplementation markedly decreased H3K4me2 enrichment at *ANGPT2* and *MMP7* promoters in both ALDH6A1-deficient and MMA-treated cells (Fig. 7, Q and R).

In summary, MMA inhibits the demethylation activity of KDM5C by competing with α-KG for binding to KDM5C, leading to the enrichment of H3K4me2 at the promoters of invasion-related genes, ultimately promoting gastric cancer cell invasion and migration.

### Targeting ALDH6A1-mediated valine metabolic remodeling suppresses gastric cancer liver metastasis in vivo

**Fig. 8.**
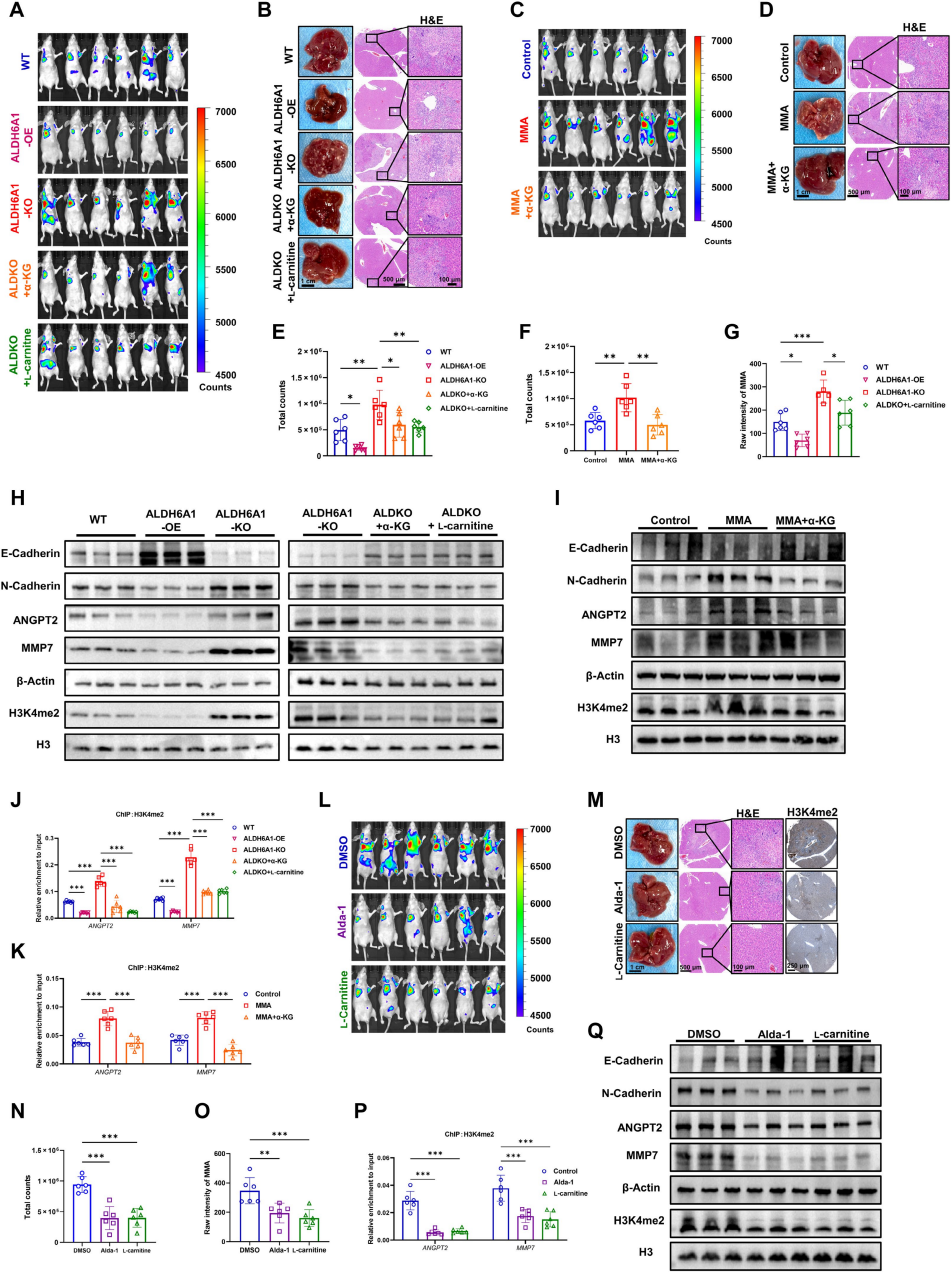
In vivo targeting of the ALDH6A1-MMA metabolic axis reduces liver metastasis of gastric cancer. (**A** and **B**) Representative bioluminescence images (A), gross liver morphology, and hematoxylin and eosin (H&E) staining (B) from mice injected with gastric cancer cells of the indicated genotypes or treatments, including WT, ALDH6A1-OE, ALDH6A1-KO, ALDH6A1-KO and dm-α-KG, and ALDH6A1-KO and l-carnitine. Boxed regions indicate higher-magnification views. (**C** and **D**) Representative bioluminescence images (C), gross liver morphology, and H&E staining (D) from mice injected with control or MMA-treated cells with or without dm-α-KG. (**E** and **F**) Quantification of total bioluminescence signals from (A) and (C), respectively (*n* = 6). (**G**) LC-MS/MS measurement of circulating MMA in mouse serum from the indicated groups (*n* = 5 to 6). (**H** and **I**) Western blot analysis of EMT/invasion-associated markers and H3K4me2 in metastatic tissues from the groups in (A) and (C), respectively. (**J** and **K**) ChIP-qPCR analysis showing reduced H3K4me2 enrichment at the *ANGPT2* and *MMP7* promoters after dm-α-KG or l-carnitine treatment in ALDH6A1-KO (J) and MMA-treated (K) liver metastases. (**L** to **N**) Representative IVIS images (L), gross liver morphology, H&E staining, H3K4me2 IHC (M), and photon flux quantification (N) from mice treated with Alda-1 or l-carnitine after splenic implantation of WT AGS cells (*n* = 6 per group). DMSO, dimethyl sulfoxide. (**O**) LC-MS/MS measurement of serum MMA after Alda-1 or l-carnitine treatment (*n* = 6). (**P**) ChIP-qPCR analysis of H3K4me2 enrichment at *ANGPT2* and *MMP7* promoters in metastatic tumors (*n* = 6). (**Q**) Western blot analysis of EMT markers and H3K4me2 in liver metastases. Data are means ± SD. Statistical significance was determined by the one-way ANOVA with post hoc multiple comparisons. *P < 0.05, **P < 0.01, and ***P < 0.001.

l-carnitine is an essential carrier for mitochondrial acyl group transport and organic acid handling. By forming acylcarnitine conjugates, it facilitates the transfer of acyl moieties for mitochondrial metabolism and, in the context of organic acid overload, promotes systemic detoxification by enabling conversion to excretable acylcarnitines that can be cleared through the circulation and the liver-kidney axis. This “buffering and clearance” function has been used clinically to reduce the burden of accumulated organic acids in metabolic disorders (*30*). To investigate the molecular mechanism by which low ALDH6A1 expression leads to MMA accumulation in gastric cancer cells and regulates H3K4me2, an invasion-related marker, we further evaluated the ALDH6A1-MMA-H3K4me2 axis in vivo. An intrasplenic injection model in BALB/c-nu nude mice was established to induce gastric cancer liver metastasis, and tumor progression was monitored in real time using the IVIS system. Compared with mice injected with WT AGS cells, ALDH6A1-OE significantly reduced hepatic fluorescence signals, whereas ALDH6A1 deletion markedly enhanced them; notably, the elevated metastatic signal in the ALDH6A1-deleted group was significantly attenuated by intraperitoneal administration of either dimethyl α-ketoglutarate (dm-α-KG) or l-carnitine (Fig. 8, A and B). Similarly, exogenous MMA enhanced liver fluorescence, whereas cotreatment with dm-α-KG largely reversed this effect (Fig. 8, C and D). Quantitative analysis showed that both ALDH6A1-KO and MMA treatment significantly increased the number of liver metastases, whereas ALDH6A1-OE, dm-α-KG, and l-carnitine administration markedly reduced metastatic foci (Fig. 8, E and F). In parallel, we measured serum MMA levels in these groups and found that ALDH6A1-KO increased circulating MMA levels, whereas ALDH6A1-OE or l-carnitine treatment also reduced serum MMA levels (Fig. 8G). Western blotting confirmed that invasive markers and H3K4me2 levels within liver metastases were elevated following ALDH6A1 deletion or MMA accumulation, whereas dm-α-KG or l-carnitine treatment attenuated these pro-invasive effects (Fig. 8, H and I). ChIP-qPCR further demonstrated that H3K4me2 enrichment at the *ANGPT2* and *MMP7* promoters in hepatic metastases was significantly increased in ALDH6A1-KO or MMA-treated mice, and dm-α-KG or l-carnitine interventions restored the levels to baseline (Fig. 8, J and K).

In our study, MMA accumulation in gastric cancer cells resulted from reduced ALDH6A1 expression and consequent loss of enzymatic activity. Alda-1, an agonist of ALDH6A1, has been shown to enhance its enzymatic function (*31*). To evaluate whether the ALDH6A1-MMA axis could serve as a therapeutic target for gastric cancer liver metastasis, we administered Alda-1 and l-carnitine via intraperitoneal injection in a nude mouse model of gastric cancer liver metastasis. The results showed that both Alda-1 and l-carnitine effectively suppressed the formation of gastric cancer liver metastases (Fig. 8, L to N). These therapeutic effects were accompanied by reduced serum MMA levels (Fig. 8O), decreased H3K4me2 enrichment at the promoters of the invasion-associated genes *ANGPT2* and *MMP7* (Fig. 8P), and an overall attenuation of the invasive phenotype together with reduced H3K4me2 levels in metastatic tumor cells (Fig. 8Q).

Collectively, these in vivo results indicate that either enhancing ALDH6A1 activity or promoting MMA excretion effectively suppresses gastric cancer liver metastasis, highlighting the ALDH6A1-MMA metabolic axis as a potential therapeutic target for metastatic gastric cancer.

## DISCUSSION

Metabolic reprogramming enables tumor cells to withstand multiple stresses, including rapid proliferation, hypoxia, and immune surveillance, and is accompanied not only by classical aerobic glycolysis but also by enhanced BCAA catabolism, active TCA cycle replenishment, and up-regulated fatty acid β-oxidation. These metabolic adaptations supply carbon sources and reducing equivalents for biosynthesis while generating metabolites that directly regulate chromatin states (*32*, *33*). α-KG–dependent dioxygenases, including the JMJD and TET families, require α-KG, Fe^2+^, and O_2_ to cocatalyze demethylation/hydroxylation, whereas TCA intermediates such as succinate, fumarate, and 2-hydroxyglutarate can competitively inhibit α-KG binding, resulting in increased histone or DNA methylation and activation of invasion-associated genes (*34*). Therefore, metabolite-epigenetic coupling is recognized as a key mechanism driving tumor phenotypic plasticity.

ALDH6A1, a member of the mitochondrial aldehyde dehydrogenase family, catalyzes the conversion of MMA semialdehyde to propionyl-CoA in the valine catabolic pathway. Previous studies have reported that down-regulation of ALDH6A1 in hepatocellular carcinoma alleviates mitochondrial respiratory inhibition by propionyl-CoA and promotes tumor progression through modulation of cellular respiration (*14*). MMA, a diagnostic marker of vitamin B_12_ deficiency, is also elevated in the circulation during aging and can promote tumor aggressiveness by inducing EMT and invasive programs in cancer cells (*16*, *18*). In addition, aggressive cancer cells can produce MMA; together, tumor-derived and aging-associated MMA can accumulate in the tumor stroma and remodel the microenvironment by activating fibroblasts through reactive oxygen species (ROS)–dependent and canonical/noncanonical transforming growth factor–β (TGFβ) signaling, thereby promoting metastatic progression (*17*). MMA also directly impairs mitochondrial function in CD8^+^ T cells in the tumor microenvironment and promotes the exhaustion phenotype, substantially weakening antitumor immunity (*35*). MMA has been implicated as a prometastatic metabolite in multiple tumor contexts; however, accumulating evidence suggests that the downstream EMT wiring of MMA is context dependent. In some settings, MMA has been reported to engage a transcription factor–centered EMT program, exemplified by SOX4-linked induction of EMT traits and metastatic competence, potentially coupled to autocrine TGFβ signaling and microenvironmental contributions (*17*, *18*). In parallel, MMA may also influence EMT indirectly through the tumor microenvironment, for instance, by remodeling stromal states [e.g., cancer-associated fibroblast (CAF) activation] and paracrine signaling that amplifies EMT programs (*17*). By contrast, our data in gastric cancer support a distinct dominant route: MMA (or ALDH6A1 loss–driven MMA accumulation) antagonizes α-KG–dependent chromatin enzymes, leading to functional inhibition of KDM5C and accumulation of H3K4me2 at promoters. The relative contribution of these routes is likely shaped by tumor lineage, baseline EMT state, stromal signaling, exposure mode (systemic versus cell-intrinsic MMA accumulation), and the local α-KG/MMA balance.

Our CRISPR-Cas9 invasion screening, using metabolic libraries, revealed that persistent low expression of the valine degrading rate-limiting enzyme ALDH6A1 in gastric cancer leads to abnormal accumulation of the metabolic by-product MMA. Structurally similar to succinate, MMA can directly occupy the α-KG–binding pocket of KDM5C, inhibit its demethylase activity, and thus increase H3K4me2 levels. In addition, MMA impairs mitochondrial function and induces exhaustion of CD8^+^ T cells while also activating fibroblasts, collectively indicating that the ALDH6A1-MMA axis acts as a multidimensional prometastatic driver that spans tumor, stromal, and immune compartments.

Although we have linked the ALDH6A1-MMA-KDM5C-H3K4me2 axis to liver metastasis in gastric cancer, several important questions remain. First, it is still unclear whether MMA simultaneously regulates other α-KG–dependent enzymes, which warrants systematic investigation. Second, the sustained down-regulation of ALDH6A1 may be influenced by multiple factors, including replication stress, hypoxia, and inflammatory signals, yet the upstream regulatory network remains largely unresolved. Last, both clinical samples and animal models need to be analyzed on a larger, multicenter scale to validate MMA as a prognostic biomarker or therapeutic monitoring indicator. Key future directions include mapping the global inhibitory profile of MMA on α-KG–dependent enzymes, elucidating how the tumor microenvironment interacts with ALDH6A1 transcriptional regulation, and evaluating the potential of combining Alda-1 or l-carnitine with immunotherapy or targeted therapies to enhance antimetastatic efficacy. An important remaining question is what drives progressive ALDH6A1 suppression during gastric cancer evolution. Although our patient data support clinical relevance, ALDH6A1 down-regulation is likely context dependent and may reflect multiple upstream inputs, including epigenetic silencing (*36*), and microenvironmental stresses (hypoxia, inflammatory cues, and nutrient limitation) that reprogram transcription and may select for states in which MMA accumulation confers advantage (*37*). Posttranscriptional regulation (e.g., microRNAs) and copy number–driven or transcription factor–driven effects may also contribute (*38*). Defining the dominant upstream mechanism will require stage-resolved integration of methylation/accessibility, transcriptional regulation, and microenvironmental perturbations, which will be an important direction for future work.

In conclusion, this study revealed the key metabolic factors of liver metastasis in gastric cancer from the perspective of metabolite-epigenetic modification and proposed a feasible strategy to restore the demethylation activity of KDM5C and inhibit H3K4me2 enrichment by activating ALDH6A1 or accelerating MMA excretion.

## MATERIALS AND METHODS

### Cell lines and culture conditions

The human gastric mucosal epithelial cell line GES-1; the gastric cancer cell lines AGS, HGC-27, MKN28, MKN45, and KATO-III; and the human embryonic kidney epithelial cell line HEK-293T were obtained from the China Center for Type Culture Collection. All cells were cultured at 37°C in a humidified incubator with 5% CO_2_ in Dulbecco’s modified Eagle’s medium (DMEM; Gibco) supplemented with 10% (v/v) fetal bovine serum (FBS; Gibco) and 1% penicillin-streptomycin (Gibco).

### Samples from patients with gastric cancer

Human samples (including gastric cancer–related specimens and/or serum, as applicable) were collected at the Zhongnan Hospital of Wuhan University. The study protocol was reviewed and approved by the Medical Ethics Committee, Zhongnan Hospital of Wuhan University (approval no. 2023211K). Written informed consent was obtained from all participants before sample collection, and the study was conducted in accordance with the Declaration of Helsinki and institutional guidelines. Clinicopathological characteristics are summarized in table S1. Tissue samples designated for RNA or protein analyses were snap frozen in liquid nitrogen and stored at −80°C, whereas specimens for IHC were fixed in 4% paraformaldehyde and embedded in paraffin. Whole-blood samples were centrifuged, and the resulting sera were aliquoted and stored at −80°C.

### CRISPR-Cas9 metabolic library invasion screen

A pooled lentiviral library was constructed in the lentiCRISPR v2 backbone, encoding Cas9 and 6896 single guide RNAs (sgRNAs) targeting 1724 human metabolic genes (four sgRNAs per gene). AGS cells (4 × 10^6^) were transduced at an MOI of <0.3, achieving ~500-fold coverage per sgRNA. Forty-eight hours after transduction, cells were selected with puromycin (1 μg/ml) for 3 days. Before each screening round, we confirmed the presence of at least 4 × 10^6^ cells (500× coverage). Invasion selection was performed using Matrigel-coated Transwell chambers. Cells that migrated through the membrane were collected, expanded, and subjected to subsequent rounds of selection for five consecutive rounds. Genomic DNA was extracted from the parental population (round 0) and from rounds 2, 4, and 5, followed by PCR amplification of sgRNA cassettes and deep sequencing on an Illumina platform. SgRNA enrichment and depletion were quantified using MAGeCK, and dynamic changes across screening rounds were characterized by *K*-means clustering.

### Gene expression analysis in public gastric cancer datasets

To validate the CRISPR screening results, we examined the expression of candidate genes across human gastric cancer cohorts. Normalized transcriptomic data for gastric adenocarcinoma (STAD; 375 tumors and 32 nontumor controls) were obtained from TCGA. In addition, five independent RNA-seq datasets (GSE122796, GSE174237, GSE179252, GSE193453, and GSE248612) were retrieved from the GEO. For each dataset, raw count data were used for differential expression analysis with DESeq2, whereas log_2_ CPM-transformed values were used for visualization. Gene expression changes were reported as log_2_FC together with the corresponding adjusted *P* values. Genes with |log_2_FC| ≥ 1 and *P* < 0.05 were defined as differentially expressed.

### Multiplex immunohistochemistry

Paraffin-embedded tissue sections were baked at 60°C for 2 hours, deparaffinized in xylene, and rehydrated through a graded ethanol series. Antigen retrieval was performed in EDTA buffer under high-pressure/boiling conditions, followed by quenching of endogenous peroxidase activity with 3% H_2_O_2_ for 10 min. Sections were blocked with 3% bovine serum albumin for 30 min at room temperature and incubated overnight at 4°C with the primary antibody. After washing with phosphate-buffered saline (PBS), slides were incubated with the corresponding horseradish peroxidase (HRP)–conjugated secondary antibody for 2 hours at room temperature and developed using tyramide signal amplification (TSA) fluorophore for 10 min. The HRP complex was then inactivated, and the cycle—from antigen retrieval through TSA development—was repeated for each additional antibody until all markers were sequentially labeled. Last, nuclei were counterstained with 4′,6-diamidino-2-phenylindole (DAPI), and sections were mounted with antifade medium before imaging. Quantification of mIHC signals was performed using QuPath. Whole-slide images were imported into QuPath, regions of interest were annotated, and cells were segmented on the basis of DAPI. Marker positivity and fluorescence intensity were quantified using standardized thresholds applied uniformly across samples, and results were reported as mean fluorescence intensity per cell.

### Generation of stable overexpression and knockout cell lines

Full-length human cDNAs for *ALDH6A1* (WT, NM_005589.4; catalytic MUT, c.514T>C), *KDM5A* (NM_001042603.3), *KDM5B* (NM_001314042.2), and *KDM5C* (NM_004187.5) were PCR amplified and cloned into the pHAGE-3×Flag-MCS lentiviral vector. For virus production, HEK-293T cells were cotransfected with the expression construct, psPAX2, and pMD2.G using Lipofectamine 3000 (Thermo Fisher Scientific). Viral supernatants were collected at 48 and 72 hours, filtered through a 0.45 μm membrane, and used to transduce target cells in the presence of polybrene (8 μg/ml). Stably infected cells were selected with puromycin (1 μg/ml) for 3 days and subsequently maintained under antibiotic selection. CRISPR-mediated ALDH6A1-KO was performed using four independent sgRNAs targeting ALDH6A1, which were designed using the MIT CRISPR design tool and cloned into lentiCRISPR v2. Lentivirus production, infection, and puromycin selection were carried out as described above. Single-cell clones were isolated by limiting dilution and screened for biallelic disruption using PCR, Sanger sequencing, and immunoblotting or RT-qPCR. Primer pairs used for cDNA amplification are listed in table S2, and sgRNA target sequences are provided in table S3. All gain-of-function and loss-of-function cell lines were validated using Western blotting or RT-qPCR before use.

### Cell culture treatments

To investigate the effects of MMA on gastric cancer cells, AGS and HGC-27 cells were treated with MMA (MACKLIN, catalog no. M824234) at final concentrations of 0, 2, 4, 6, 8, or 10 mM for 10 days, as previously described (*18*). The culture medium was supplemented with 50 mM Hepes (Biosharp, catalog no. BL1061A) to maintain pH stability throughout the treatment period. For rescue experiments, α-KG (MedChemExpress, catalog no. HY-W013636) was added to MMA-treated cultures at a final concentration of 5 mM and coincubated for the same 10-day period. The medium containing the respective metabolites was refreshed every 48 hours.

### In vitro invasion and migration assay

AGS and HGC-27 cells in the logarithmic growth phase were detached using trypsin, washed, and resuspended in serum-free DMEM at a concentration of 5 × 10^5^ cells per 100 μl. For migration assays, 600 μl of DMEM containing 10% FBS was added to the lower chamber of 24-well Transwell inserts (8-μm pore size; NEST, catalog no. 725331). A 100-μl cell suspension was then added to the upper chamber and incubated for 36 hours at 37°C. Nonmigrated cells on the upper surface were removed with a cotton swab, whereas cells that had migrated to the underside of the membrane were fixed with 4% paraformaldehyde, stained with crystal violet, and counted under a microscope. For invasion assays, inserts were precoated on the upper surface with Matrigel (Corning, catalog no. 356234) diluted 1:10 in cold serum-free DMEM and allowed to polymerize at 37°C for 1 hour. Cells were seeded and processed as described above. Results are presented as the mean number of migrated or invaded cells from at least three independent experiments.

### RNA-seq and analysis

Total RNA-seq was performed at the Analysis and Testing Center of the Institute of Hydrobiology, Chinese Academy of Sciences. Polyadenylate [Poly(A)]–enriched RNA was amplified to generate cDNA libraries, which were sequenced on an Illumina platform. Raw FASTQ reads were quality trimmed using Trimmomatic and aligned to the human reference genome (GRCh38.p14) with HISAT2 (v2.2.1). Gene-level counts were generated using featureCounts, and differential expression analysis was performed with DESeq2 (v1.34.0). Differentially expressed genes were further subjected to *K*-means unsupervised clustering, and pathway enrichment analysis for each cluster was conducted using the KEGG database or Gene Ontology (GO) biological process terms.

### Untargeted metabolomics

Cells were collected separately, centrifuged to remove the debris, snap frozen in liquid nitrogen, stored at −80°C, and shipped on dry ice to Biomarker Technologies (Beijing, China). For untargeted metabolomics, intracellular metabolites were extracted with a cold aqueous organic solvent containing internal standards, proteins were precipitated, and supernatants were analyzed by ultra-high-performance liquid chromatography (UHPLC) coupled to high-resolution MS in both electrospray ionization–positive (ESI+) and ESI− modes with data-dependent MS/MS. Raw data were processed for peak detection, features were filtered using quality control (QC) reproducibility and blank subtraction, metabolites were annotated by accurate mass/retention time and MS/MS spectral matching against public and in-house libraries, and signals were normalized to cell number before statistical analysis with multiple-testing correction.

### Liquid chromatography–tandem mass spectrometry

Targeted quantification of pyruvic acid, lactic acid, MMA, succinic acid, α-ketoglutaric acid, and fumaric acid was performed by LC-MS/MS. Cells were aliquoted into 2-ml tubes, extracted with 1 ml of 70% methanol, homogenized (25 Hz, 10 min) and sonicated on ice (10 min), and centrifuged (4°C, 12,000 rpm, 10 min), and the supernatant (500 μl) was filtered (0.22 μm) for analysis. A mixed standard stock (10 mg/liter) was prepared from neat standards and serially diluted to generate calibration curves. Separation was performed on a Waters ACQUITY I-Class UPLC with an ACQUITY HSS T3 C18 column (1.8 μm, 2.1 mm by 100 mm) (30°C; autosampler: 10°C; injection: 2 μl) using A: 0.1% formic acid in water and B: methanol. Detection used a SCIEX QTRAP 6500+ (IonDrive Turbo V) in multiple reaction monitoring (MRM) mode with optimized transitions; data were acquired and quantified in Analyst v1.7.2/SCIEX OS v2.0.1. Calibration used weighted 1/*x* regression; limit of detection (LOD) and lower limit of quantification (LLOQ) were defined at signal-to-noise ratio (S/N ratio) = 3/10, and QC and accuracy were monitored by relative SD and spike recovery, with final concentrations calculated using dilution factors and normalized to sample mass/volume as appropriate. For mouse serum MMA quantification, serum samples were thawed on ice, mixed with cold methanol containing internal standards, vortexed, and centrifuged to remove precipitated proteins. The resulting supernatants were subjected to LC-MS/MS analysis using the same MRM-based quantification workflow.

### [U-^13^C_5_]valine isotope tracing assay

WT and ALDH6A1-KO AGS cells were incubated in l-valine–free medium (Servicebio, YC-1052) and then exposed to l-valine-^13^C_5_ (MCE, HY-N0717S6) at 10 mg/500 ml (~164 μM) for 6 hours. Cells were rapidly washed with ice-cold PBS, and intracellular metabolites were extracted using prechilled [methanol/acetonitrile/water]. Extracts were analyzed by LC-MS/MS. Isotopolog peak areas were normalized to the total signal of each metabolite to generate mass isotopolog distributions. Total labeled fraction was calculated as the sum of all labeled isotopologs excluding M+0. Labeling of valine, α-ketoisovaleric acid, and MMA was used to evaluate valine catabolic flux and carbon diversion toward MMA accumulation.

### Enzyme-linked immunosorbent assay

MMA concentrations in patient serum were quantified using a competitive enzyme-linked immunosorbent assay (ELISA) kit (Meimian, catalog no. MM-92661101) according to the manufacturer’s instructions. Briefly, 50 μl of the sample or MMA standard was added in duplicate to antibody-coated microplate wells, followed by 50 μl of the HRP-conjugated reagent. After incubation at 37°C for 60 min, wells were washed five times and developed for 15 min with 3,3′,5,5′-tetramethylbenzidine substrate, and the reaction was stopped with 2 M H_2_SO_4_. Absorbance was measured at 450 nm using a SpectraMax iD5 microplate reader (Molecular Devices). MMA concentrations were calculated from a four-parameter logistic standard curve and normalized to sample volume.

### Chromatin immunoprecipitation

ChIP was performed using the SimpleChIP Plus Sonication Chromatin IP kit (Cell Signaling Technology, catalog no. 56383) according to the manufacturer’s instructions. Briefly, cells (2 × 10^7^ per IP) or pulverized frozen tissues (~50 mg) were cross-linked in 1% formaldehyde for 10 min at 22°C and quenched with 125 mM glycine for 5 min. Nuclei were lysed in SDS buffer, and chromatin was sheared by sonication (Covaris M220; 10% duty cycle, 200 cycles per burst, 10 min) to an average fragment size of 200 to 500 base pairs (bp). After clarification, 2% of each sample was reserved as input, and the remainder was incubated overnight at 4°C with 5 μg of anti-H3K4me2 antibody (Abcam, ab32356) prebound to protein G magnetic beads. Immunocomplexes were washed, eluted, and decross-linked at 65°C for 2 hours, and DNA was purified using the spin columns provided in the kit. For ChIP-qPCR, eluates were analyzed in triplicate with SYBR Green Master Mix (Toyobo) on a CFX Connect instrument (Bio-Rad); primer sequences are listed in table S4. Results are expressed as the percent input. For ChIP-seq, libraries were prepared using the NEBNext Ultra II kit and sequenced (2 × 150 bp) on an Illumina NovaSeq 6000 at the Institute of Hydrobiology, Chinese Academy of Sciences. Reads were aligned to GRCh38 with Bowtie2, peaks were called using MACS2, and data were visualized in the Integrative Genomics Viewer and the UCSC Genome Browser.

### HMT and HDM activity assays

Global H3K4 HMT and HDM activities were measured using the EpiQuik HMT (H3K4-specific) Activity/Inhibition Assay Kit (EpigenTek, catalog no. P-3002) and the EpiQuik HDM (H3K4-specific) Activity/Inhibition Assay Kit (EpigenTek, catalog no. P-3074), respectively, following the manufacturer’s instructions. Nuclear extracts (10 μg of protein per reaction) were incubated with the immobilized H3K4 peptide substrate at 37°C for 60 min. In the HMT assay, the unmodified substrate remaining after methyl group transfer was detected colorimetrically using an HRP-conjugated antibody and measured at 450 nm on a SpectraMax iD5 plate reader. In the HDM assay, the demethylated product was detected fluorometrically (excitation/emission: 530/590 nm). Enzyme activity (arbitrary units/mg protein) was calculated from a standard curve generated with kit calibrators and normalized to the amount of input nuclear protein. All samples were analyzed in technical triplicate.

### Molecular docking

Three-dimensional structures of MMA (CID: 487) and α-KG (CID: 51) were retrieved from PubChem and energy minimized using AutoDock Tools 1.5.7. The full-length human KDM5C structure was modeled with AlphaFold 3, achieving a confidence score of >0.80 for the catalytic domain. Docking calculations were carried out in AutoDock 4 using the Lamarckian genetic algorithm, following the procedure described previously (*39*), and the final results were visualized using PyMOL 3.1.

### Protein expression and purification

A cDNA fragment encoding the catalytic domain of human KDM5C was cloned into pET-26b(+) to generate a C-terminal His-tag fusion. The construct was transformed into *Escherichia coli* BL21(DE3). Cultures were grown at 37°C to an OD_600_ (optical density at 600 nm) of 0.6, chilled to 16°C, and induced with 0.2 mM isopropyl-β-d-thiogalactopyranoside (IPTG) for 24 hours. Cells were harvested, resuspended in lysis buffer [50 mM tris-HCl (pH 8.0), 300 mM NaCl, 10 mM imidazole, and 1 mM phenylmethylsulfonyl fluoride (PMSF)], and lysed by sonication. After centrifugation (20,000*g*, 30 min, 4°C), the supernatant was loaded onto a HisTrap HP column and eluted with a 10 to 250 mM imidazole gradient on an ÄKTA Avant system (Cytiva). Peak fractions were pooled, concentrated using a 10-kDa cutoff ultrafiltration device, and further purified by size exclusion chromatography in 20 mM tris-HCl at pH 8.0, 150 mM NaCl, and 5% (v/v) glycerol. Purified protein was flash frozen in liquid nitrogen and stored at −80°C.

### Isothermal titration calorimetry

The binding of MMA and α-KG to the KDM5C protein was measured using a Nano ITC system (TA Instruments, USA). KDM5C (200 μM) and the ligands MMA and α-KG (each 10 mM) were prepared in the same buffer. Fifty microliters of ligand solution was titrated into the protein solution in the ITC cuvette at 37°C, with 2.5 μl per drop and 120-s intervals between drops. The binding parameters, including the association constant (*K*_a_), dissociation constant (*K*_d_), enthalpy change (Δ*H*), and entropy change (Δ*S*), were determined by analyzing the resulting thermograms.

### Reverse transcription qPCR

Total RNA from cultured cells or frozen tissues was extracted using TRIzol reagent (Sigma-Aldrich, catalog no. T9424) following the manufacturer’s instructions. One microgram of RNA was reverse transcribed using the ReverTra Ace qPCR RT kit (TOYOBO, catalog no. FSQ-101). qPCR was performed in triplicate using THUNDERBIRD SYBR qPCR Mix (TOYOBO, catalog no. QPS-201) on a CFX96 Touch or CFX384 Touch Real-Time PCR System (Bio-Rad) and, for selected assays, on a LightCycler 96 (Roche). Cycling conditions included 95°C for 1 min, followed by 40 cycles of 95°C for 15 s and 60°C for 60 s. Melt-curve analysis confirmed single-product amplification. Relative mRNA levels were calculated using the 2^−ΔΔCt^ method with β-actin as the internal control. Primer sequences are listed in table S5.

### Western blotting

Cells or pulverized frozen tissues were lysed in ice-cold radioimmunoprecipitation assay (RIPA) buffer supplemented with protease and phosphatase inhibitors (Roche). Protein concentrations were determined using the BCA assay (Thermo Fisher Scientific, catalog no. A55864). Equal amounts of lysate (20 to 40 μg) were separated using SDS–polyacrylamide gel electrophoresis (PAGE) and transferred to polyvinylidene difluoride (PVDF) membranes. Membranes were blocked in 5% (w/v) nonfat milk in TBST for 1 hour at 22°C and then incubated overnight at 4°C with primary antibodies (table S6), followed by species-matched HRP-conjugated secondary antibodies for 1 hour at room temperature. Protein bands were visualized with ECL substrate (Epizyme, catalog no. SQ201) on a Tanon 5200 chemiluminescence imager and quantified using ImageJ.

### Mouse model

All animal procedures were approved by the Animal Ethics Committee of Zhongnan Hospital of Wuhan University (ZN2024006). Female BALB/c nude mice (4 weeks old) were maintained under specific pathogen–free (SPF) conditions (25°C, 12-hour light/ 12-hour dark cycle). For splenic injection, 2.5 × 10^6^ luciferase-labeled AGS cells in 100 μl of PBS were slowly injected into the splenic parenchyma, and livers were collected 8 weeks later. Whole-body bioluminescence was recorded biweekly using an IVIS Spectrum system (PerkinElmer). Metabolic or pharmacological interventions were administered daily via intraperitoneal injection: MMA, 400 μg/g body weight; dm-α-KG, 200 μg/g; Alda-1, 50 μg/g; and l-carnitine, 500 μg/g. Metabolites were administered for 7 days before tumor implantation and continued thereafter, whereas Alda-1 and l-carnitine treatments began 7 days post–cell injection and continued until the end of the study.

### Statistical analysis

Data are presented as means ± SD and were plotted and analyzed using GraphPad Prism 9.0. Comparisons between two groups were performed using two-tailed Student’s *t* tests, whereas comparisons among three or more groups were performed using one-way analysis of variance (ANOVA) followed by Tukey’s post hoc test, Two-way ANOVA was used where two independent variables were analyzed. The OS was assessed using Kaplan-Meier curves and the log-rank test. All experiments were independently repeated at least three times. Statistical significance was defined as *P < 0.05, **P < 0.01, or ***P < 0.001.
