# Supplementary material for: Reprogramming of valine metabolism mediated by abnormally low ALDH6A1 expression promotes invasive metastasis of gastric cancer
Source: Sci Adv. 2026 Jul 8;12(28):eaeb2892. doi: 10.1126/sciadv.aeb2892 (PMC13344286; doi:10.1126/sciadv.aeb2892)
Supplement: Supplementary file 1 — Tables S1 to S6 Figs. S1 to S11 [file sciadv.aeb2892_sm.pdf]

Science Advances
AAAS

Supplementary Materials for

Reprogramming of valine metabolism mediated by abnormally low ALDH6A1 expression promotes invasive metastasis of gastric cancer

Jipeng Wang *et al.*

Corresponding author: Yan-Xiao Ji, jiyanxiao@whu.edu.cn; Bin Xiong, binxiong1961@whu.edu.cn; Shuyi Wang, shuyiwang@whu.edu.cn

Sci. Adv. **12**, eaeb2892 (2026)
DOI: 10.1126/sciadv.aeb2892

This PDF file includes:

Tables S1 to S6
Figs. S1 to S11

Table S1. The characteristics of gastric cancer patients (n=57)

**Characteristics**		**No. of patients (%)**
**Age (years)**	< 60	27 (47.4%)
	≥ 60	30 (52.6%)
**Gender**	Male	36 (63.2%)
	Female	21 (36.8%)
**Tumour location**	Proximal (cardia/fundus)	19 (33.3%)
	Body	26 (45.6%)
	Distal (antrum/pylorus)	12 (21.1%)
**Lauren classification**	Intestinal	28 (49.1%)
	Diffuse	22 (38.6%)
	Mixed	7 (12.3%)
**T stage**	T1	7 (12.3%)
	T2	12 (21.0%)
	T3	22 (38.6%)
	T4	16 (28.1%)
**N stage**	N0	17 (29.8%)
	N1+N2	40 (70.2%)
**M stage**	M0	46 (80.7%)
	M1	11 (19.3%)

Table S2. The primer sets for PCR amplification and subcloning

Gene	Vector	Primer sets
ALDH6A1	pHAGE-3×Flag	CGACGCGTATGGCGGCGCTATTGGCG
ALDH6A1^{T514C}	pHAGE-3×Flag	ATTCCCACCGTCTGCCTCT
KDM5A	pHAGE-3×Flag	GGATCGGGTTTAAACGGATCCATGGCGGGCGTGGG
KDM5B	pHAGE-3×Flag	GGATCGGGTTTAAACGGATCCATGGAGGCGGCCACC
KDM5C	pHAGE-3×Flag	GGATCGGGTTTAAACGGATCCATGGAGCCGGGGTCCG

Table S3. The sgRNA sequences used for CRISPR-cas9 knockout

Gene	Number	Sequence
ALDH6A1	sgRNA1	AGTTAATCACATTGGAACAA
	sgRNA2	CATCTGGCATGACTACCCCA
	sgRNA3	CCATGGGAAACATCCAAAGG
	sgRNA4	GGAACATTAAACATCATCCA

Table S4. The primer sets for quantitative ChIP-qPCR

Gene	Forward	Reverse
ANGPT2	CCAGCTTAGCACGGCAAAAA	TCCTGTAGGGGGTCACTAGC
MMP7	AATGCAGCCCTACCTGTAGC	CCTTCCACGTCCCTTAGCAG

Table S5. The primer sets for quantitative RT-PCR

Gene	Forward	Reverse
β-actin	GTCATTCCAAATATGAGATGCGT	GCTATCACCTCCCCTGTGTG
ALDH6A1	GGCAGACACTTCAGTATTAAGCC	AGAGGCAGACGGTAGGAATAAA
MMP7	GAGTGAGCTACAGTGGGAAC	CTATGACGCGGGAGTTTAACAT
ANGPT2	AGATTTTGGACCAGACCAGTGA	GGATGATGTGCTTGTCTTCCAT
KMT2A	GCCGAAAACGAGCTGTGTTT	CTCTTTTCCTCGACGGGCTT
KMT2B	GCTGTCTGGGGTAGAGGAGA	CGCATCTTCTTGCCGTGATG
SETD1A	AGGCTACTACCCCATCAGCA	CCAATGGGCTGCTTGGAGTA
KDM1A	CACCAGCCGTTCAGTTTGTG	GGCTGGGTAGTTACGGATCG
KDM5A	TAATGGAGCATGGTGTGCCTGT	CTCTGGTTTCCTCCAATCCTTCCT
KDM5B	GGGCTCACATATCAGAGGGC	TCACACAACAGTAGCCGGTC
KDM5C	CGGAACCCACAGAGGAAGAC	AGTGTATTCCCGGGTAGCCT

Table S6. Primary antibodies used for Western blotting

Target	Species	Catalog No.	Company	Dilution
β-actin	Rabbit monoclonal	AC026	ABclonal	1:100000
ALDH6A1	Mouse monoclonal	sc-271582	Santa Cruz	1:500
E-cadherin	Mouse monoclonal	60335-1-Ig	ProteinTech	1:5000
N-Cadherin	Mouse monoclonal	66219-1-Ig	ProteinTech	1:10000
Vimentin	Mouse monoclonal	60330-1-Ig	ProteinTech	1:50000
SNAI1	Mouse polyclonal	13099-1-AP	ProteinTech	1:1000
H3K4me2	Rabbit monoclonal	A22143	ABclonal	1:5000
H3K4me	Rabbit monoclonal	A22078	ABclonal	1:5000

H3K9me2	Rabbit monoclonal	A26196	ABclonal	1:1000
H3K9me3	Rabbit monoclonal	A22295	ABclonal	1:10000
H3K27me3	Rabbit monoclonal	A22396	ABclonal	1:30000
H3K36me3	Rabbit monoclonal	A20379	ABclonal	1:3000
H3	Rabbit monoclonal	A22348	ABclonal	1:10000
MMP7	Rabbit monoclonal	A20701	ABclonal	1:1000
ANGPT2	Mouse monoclonal	sc-74403	Santa Cruz	1:500

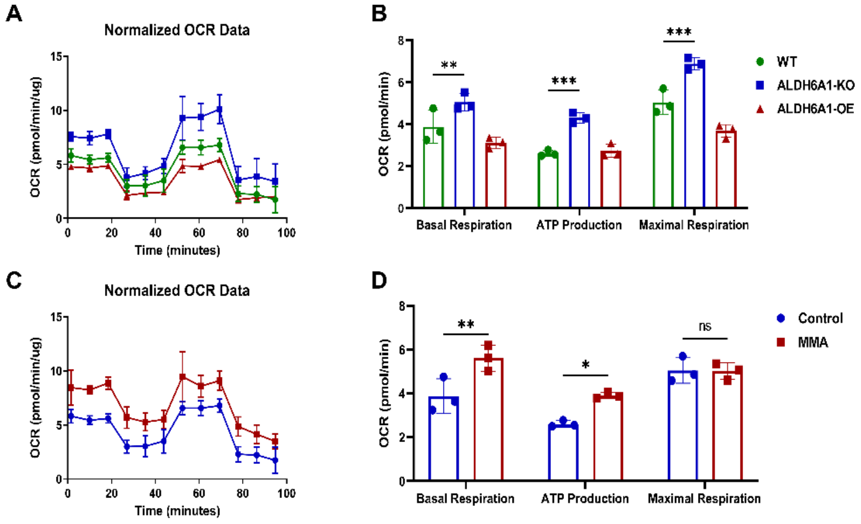

Figure S1. ALDH6A1 loss and MMA treatment enhance oxidative phosphorylation in gastric cancer cells. A Seahorse XF analysis of oxygen consumption rate (OCR) over time in AGS cells with the indicated ALDH6A1 status (WT, ALDH6A1-KO, and ALDH6A1-OE). OCR values were normalized as indicated. **B** Quantification of basal respiration, ATP-linked respiration (ATP production), and maximal respiration derived from the Seahorse assay in (A). **C** Seahorse XF OCR traces of AGS cells treated with MMA or vehicle control. **D** Quantification of basal respiration, ATP-linked respiration (ATP production), and maximal respiration derived from the Seahorse assay in (C). Data are presented as mean ± SEM. Each symbol represents an independent biological replicate. Statistical significance is indicated as ns, not significant; *$P < 0.05$, **$P < 0.01$, ***$P < 0.001$ (two-tailed unpaired Student's t test for two-group comparisons; one-way ANOVA with multiple-comparisons correction for three-group comparisons).

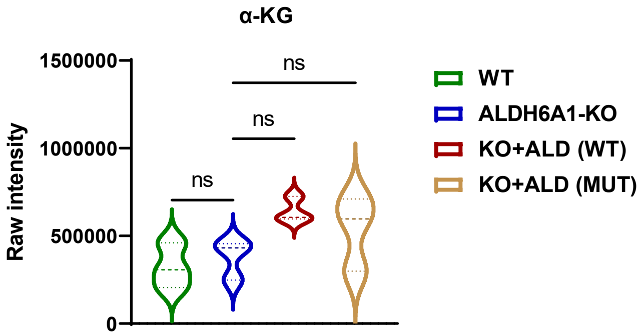

Figure S2. Changes in ALDH6A1 expression do not affect α-KG levels in gastric cancer cells. LC–MS/MS quantification of intracellular α-KG after rescue with wild-type (WT) or catalytic-dead mutant (Mut) ALDH6A1 in KO cells (n = 3 per group). Data are presented as mean ± SEM. Each symbol represents an independent biological replicate. Statistical significance is indicated as ns, not significant (two-tailed unpaired Student's t test for two-group comparisons; one-way ANOVA with multiple-comparisons correction for three-group comparisons).

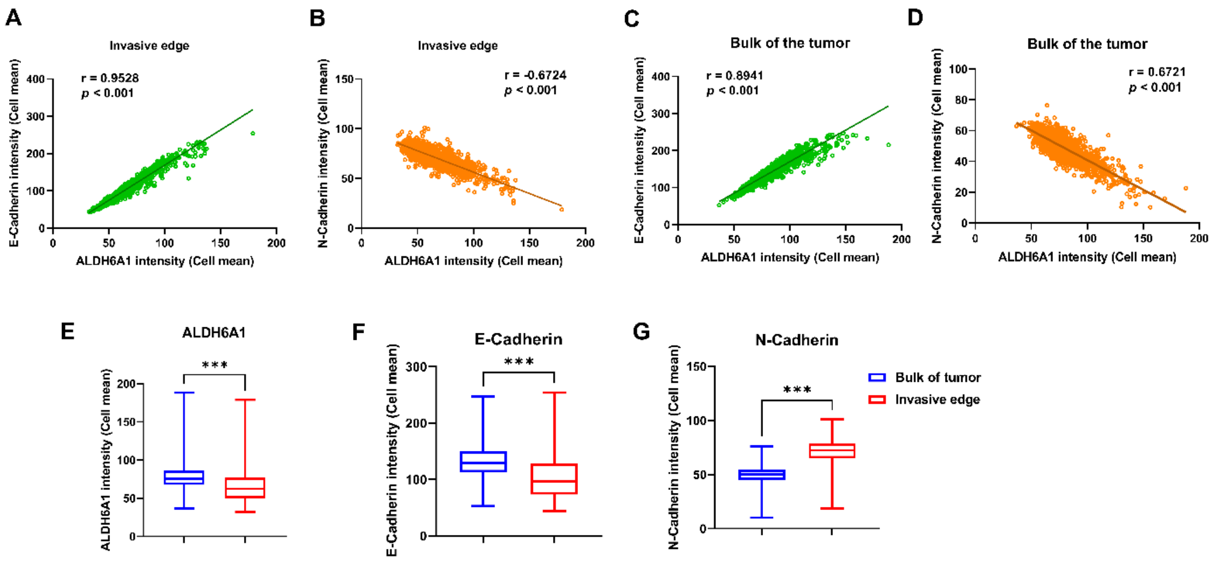

Figure S3. Cell-level correlations between ALDH6A1 and EMT markers stratified by invasive edge versus tumor bulk. A, B At the invasive edge, ALDH6A1 intensity positively correlates with E-cadherin (A) and negatively correlates with N-cadherin (B). **C, D** In the tumor bulk, ALDH6A1 intensity positively correlates with E-cadherin (C) and negatively correlates with N-cadherin (D). Correlation coefficients (r) and P values are indicated on each plot (Pearson correlation). **E to G** Comparison of ALDH6A1 (E), E-cadherin (F), and N-cadherin (G) single-cell intensities between tumor bulk and invasive edge. Box-and-whisker plots indicate median, interquartile range, and min–max. ***$P < 0.001$ (two-tailed unpaired test).

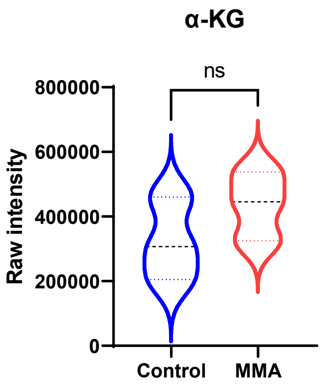

Figure S4. MMA treatment does not affect α-KG levels in gastric cancer cells. Targeted single-metabolite quantification of intracellular α-KG (oxoglutaric acid) in AGS cells treated with MMA (10 mM, 10 days) or vehicle control (n = 3 per group). Data are presented as violin plots with the median and interquartile range indicated. Statistical significance is indicated as ns, not significant (two-tailed unpaired Student's t test).

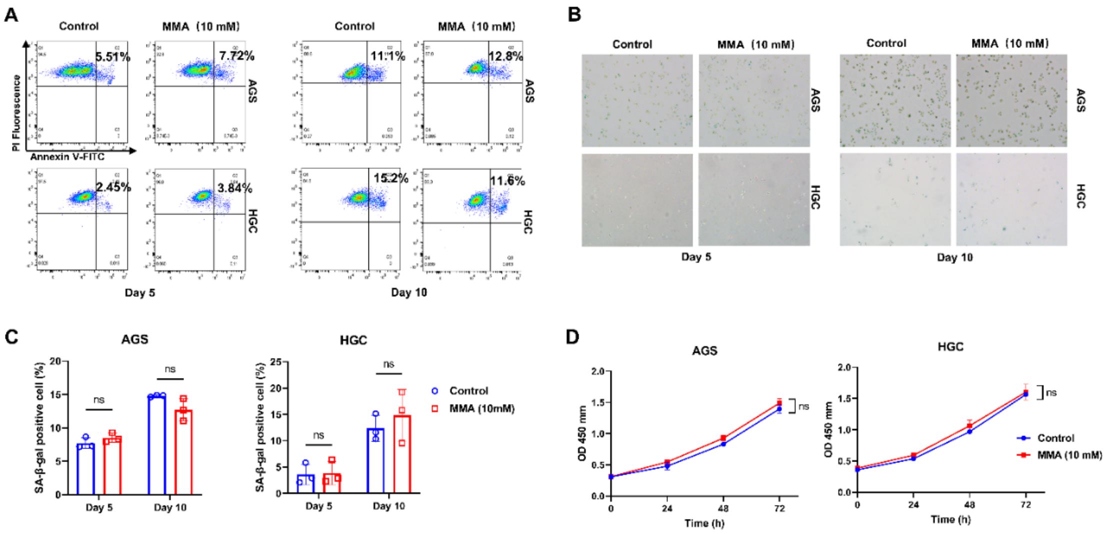

Figure S5. Prolonged MMA exposure does not induce overt cytotoxicity, senescence, or proliferation changes in gastric cancer cells. **A** Representative Annexin V–FITC/PI flow cytometry plots of AGS and HGC-27 cells treated with vehicle (Control) or MMA (10 mM) for 5 or 10 days, showing apoptotic/necrotic populations. **B** Representative SA-β-galactosidase (SA-β-Gal) staining images of AGS and HGC-27 cells after 5 or 10 days of treatment with Control or MMA (10 mM). **C** Quantification of SA-β-Gal–positive cells in AGS and HGC-27 cultures at day 5 and day 10 under Control or MMA (10 mM) treatment. **D** CCK-8 proliferation assays performed after 10 days of pre-treatment with Control or MMA (10 mM), showing comparable proliferative capacity between groups over the indicated time course. Data are presented as mean ± SEM; ns, not significant; statistical tests and sample sizes are provided in the Methods.

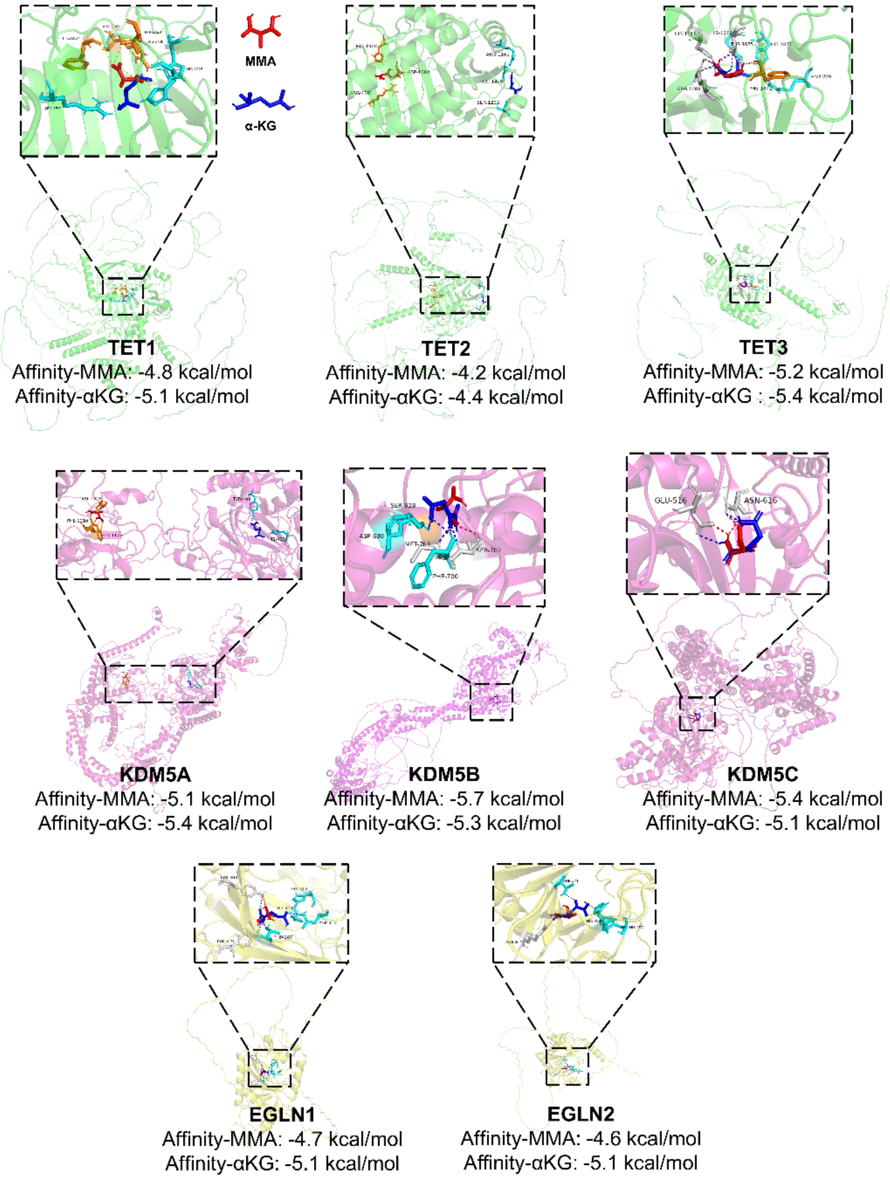

Figure S6. Predicted MMA- and α-KG–interacting residues in α-KG–dependent dioxygenases. In silico docking models of methylmalonic acid (MMA; red) and α-ketoglutarate (α-KG; blue) with TET1/2/3, KDM5A/B/C, and EGLN1/2 are shown, with enlarged views highlighting the ligand-binding pockets. Amino acid residues contacting MMA are colored orange, residues contacting α-KG are colored cyan, and residues shared by both ligands are colored gray. Predicted binding affinities (kcal/mol) for MMA and α-KG are indicated for each protein.

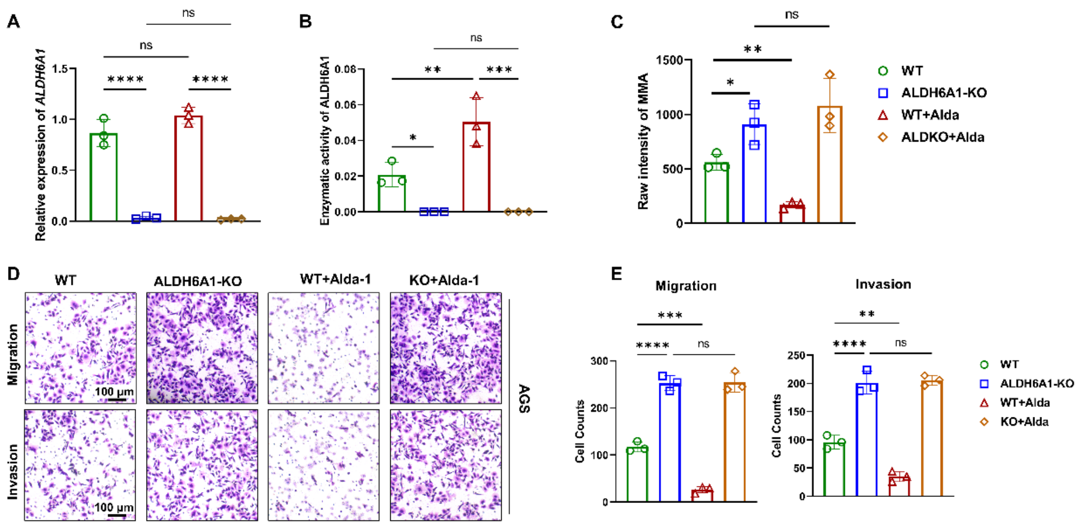

Figure S7. Alda-1 suppresses gastric cancer cell migration and invasion through an on-target, ALDH6A1-dependent mechanism. A *ALDH6A1* mRNA expression in AGS cells (WT versus ALDH6A1-KO) treated with Alda-1 or vehicle control. **B** ALDH6A1 enzymatic activity under the indicated genotypes and treatments. **C** Targeted quantification of intracellular MMA levels under the indicated genotypes and treatments. **D** Representative Transwell migration and Matrigel invasion images of AGS cells treated as indicated (scale bar, 100 μm). **E, F** Quantification of invaded (E) and migrated (F) cells from (D). Data are presented as mean ± SEM (n = 3 independent biological replicates). Statistical significance is indicated as ns, not significant; *P < 0.05, **P < 0.01, and ***P < 0.001 (two-tailed unpaired Student's t test for two-group comparisons; one-way or two-way ANOVA with multiple-comparisons correction, as appropriate).

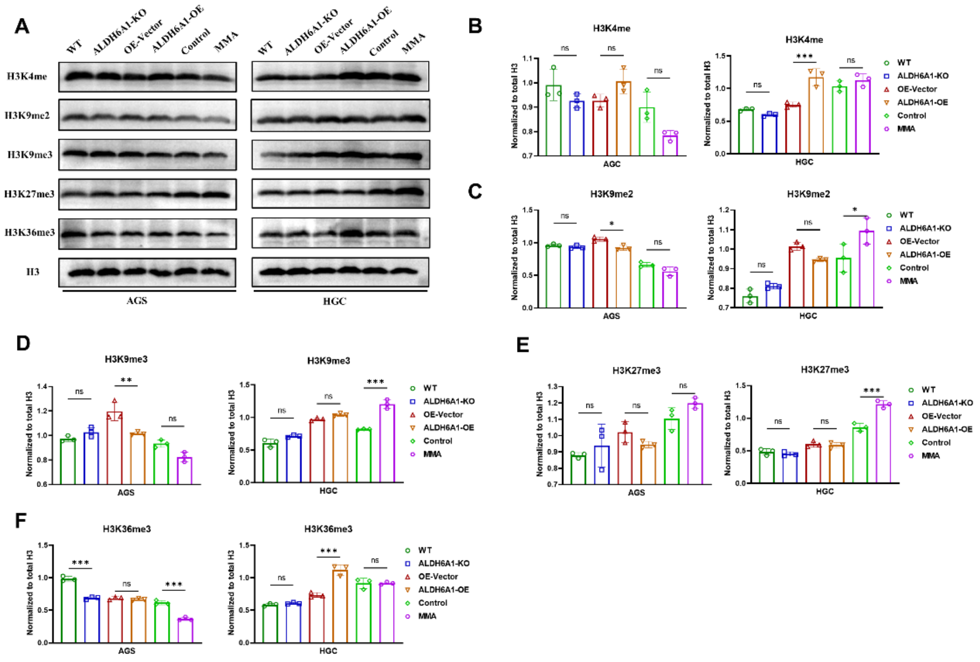

Figure S8. Global histone methylation profiling after ALDH6A1 KO/OE or MMA treatment in gastric cancer cells. A Representative immunoblots showing global histone methylation marks (H3K4me, H3K9me2, H3K9me3, H3K27me3, and H3K36me3) in AGS and HGC-27 cells under the indicated conditions (WT, ALDH6A1-KO, OE-vector, ALDH6A1-OE, vehicle control, and MMA treatment [4 mM]). **B to F** Densitometric quantification of H3K4me (B), H3K9me2 (C), H3K9me3 (D), H3K27me3 (E), and H3K36me3 (F) from (A), normalized to total H3, in AGS (left panels) and HGC-27 (right panels). Data are presented as mean ± SEM (n = 3 independent biological replicates). Statistical significance is indicated as ns, not significant; *P < 0.05, **P < 0.01, and ***P < 0.001 (one-way ANOVA with multiple-comparisons correction).

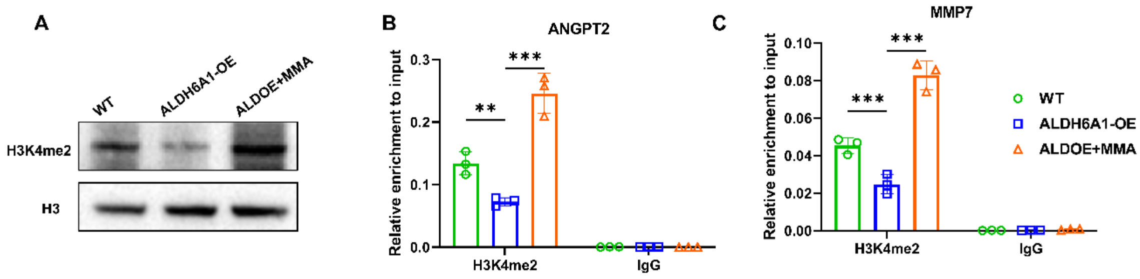

Figure S9. MMA reverses ALDH6A1 overexpression–associated reduction of H3K4me2 in gastric cancer cells. A Western blot of global H3K4me2 in AGS cells under the indicated conditions (WT, ALDH6A1 overexpression [ALDH6A1-OE], and ALDH6A1-OE treated with MMA [10 mM, 10 days]); total histone H3 serves as a loading control. **B, C** ChIP–qPCR analysis showing H3K4me2 occupancy at the promoters of *ANGPT2* (B) and *MMP7* (C) in AGS cells under the indicated conditions. IgG was used as a negative control. Data are presented as enrichment relative to input (mean ± SEM); statistical significance is indicated as $^{**}P < 0.01$ and $^{***}P < 0.001$ (one-way ANOVA with multiple-comparisons correction).

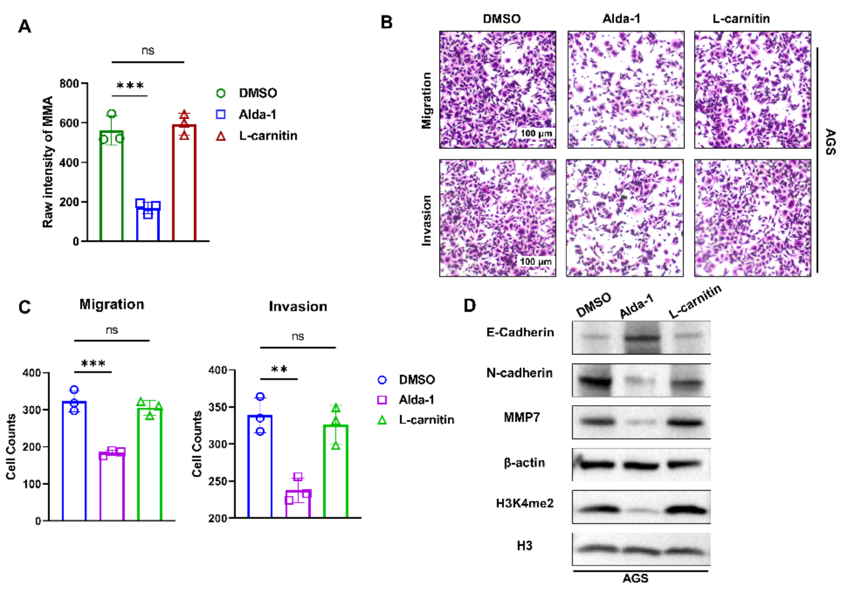

Figure S10. Distinct *in vitro* effects of Alda-1 and L-carnitine in AGS gastric cancer cells. A Targeted LC–MS/MS quantification of intracellular MMA in AGS cells treated with vehicle (DMSO), Alda-1, or L-carnitine. **B** Representative transwell images showing migration and invasion of AGS cells after treatment with DMSO, Alda-1, or L-carnitine (scale bar, 100 μm). **C** Quantification of migrated and invaded cells corresponding to (B). **D** Western blot of EMT and invasion-associated proteins (E-cadherin, N-cadherin, MMP7) and global H3K4me2 levels in AGS cells treated with DMSO, Alda-1, or L-carnitine. Data are means ± SEM; ns, not significant; $**P < 0.01$ and $***P < 0.001$ by one-way ANOVA with multiple-comparisons correction.

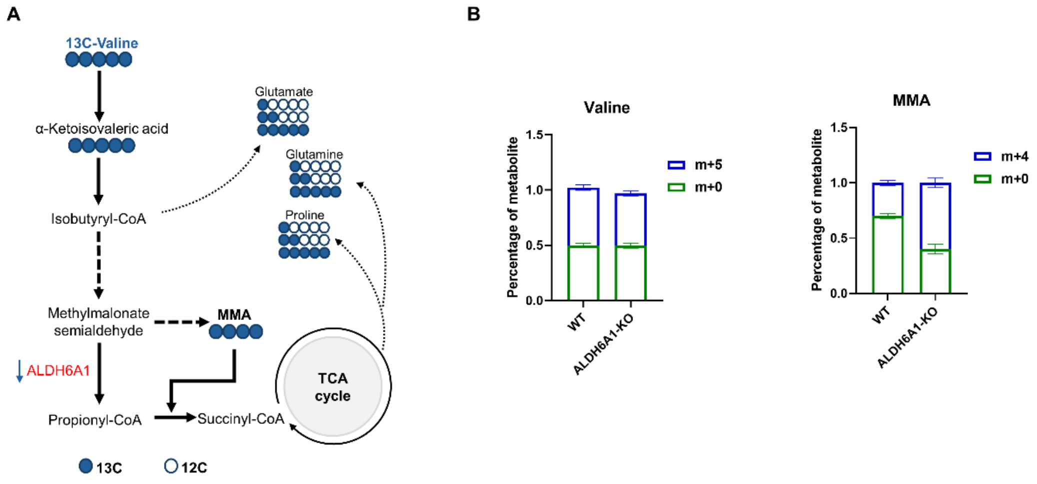

Figure S11. ALDH6A1 deficiency redirects valine-derived carbon flux toward MMA accumulation.

A Schematic illustration of [U-$^{13}C_5$] valine tracing in the valine degradation pathway. Blue circles indicate ^{13}C-labeled carbons and open circles indicate unlabeled ^{12}C. **B** Mass isotopomer distribution of valine and MMA in WT and ALDH6A1-KO AGS cells after [U-$^{13}C_5$] valine tracing.
